# Factor Structure and Validity of Composite Scores Resulting From a Computerized Cognitive Test Battery in Healthy Adults and Patients With Primary Brain Tumors

**DOI:** 10.1177/10731911241289987

**Published:** 2024-11-20

**Authors:** S.M. Boelders, E. Butterbrod, L.V.D.E. Vogelsmeier, M.M. Sitskoorn, L.L. Ong, K. Gehring

**Affiliations:** 1 Elisabeth-TweeSteden Hospital, Tilburg, The Netherlands; 2 Tilburg University, The Netherlands

**Keywords:** computerized neuropsychological testing, factor analysis, primary brain tumor, cognitive function, measurement invariance, composite scores

## Abstract

Computerized neuropsychological test batteries (CNTs), such as Central Nervous System Vital Signs (CNS VS), are increasingly used for measuring cognitive functioning, but empirical evidence of how they measure cognition is scarce. We investigated the factor structure of CNS VS using exploratory factor analyses four samples: healthy adults (*n* = 169), patients with meningioma (392), low-grade glioma (99), and high-grade glioma (247). We tested model fit and investigated measurement invariance. Differences in factor interpretation existed between healthy participants and patients. Factor structures among patient groups were approximately the same but differed in non-zero loadings. Overall, factor structures largely did not support the “clinical domains” provided by CNS VS for clinical interpretation. Confirmatory models did not have a good fit, and measurement invariance could not be established. Our results indicate that (weighted) sum scores of CNS VS results may lack validity. We recommend researchers and clinicians to use scores on individual test measures.

## Introduction

Computerized neuropsychological tests (CNTs) or test batteries are increasingly popular instruments to assess cognitive function in clinical practice and research (Gondar et al., 2021). CNTs allow for easy, standardized, and often remote test administration. In addition, they instantly provide accurate performance measures without going through manual scoring procedures that are labor-intensive and prone to errors. One popular and widely-used brief screening battery is “Central Nervous System Vital Signs” (CNS VS) (Oliveira & Brucki, 2014; Wojcik et al., 2019).

The CNS VS battery consists of seven core tests that are based on well-established pen-and-paper tasks (CNS Vital Signs, n.d.; C. Gualtieri & Johnson, 2006; Plourde et al., 2018); a visual (freely adapted from the recognition condition of the Rey Visual Design Learning test), a verbal memory recognition tasks (freely adapted from the recognition condition of the Rey Auditory Verbal Learning tests), a symbol digit coding task (based on the Wechsler Digit symbol substitution test), a shifting attention task (color-shape), a finger-tapping test, an adapted Stroop test, and a continuous performance test (C. Gualtieri & Johnson, 2006). A more detailed description of each task can be found in Supplemental Table S1. One of the main benefits of this relatively brief screening is that it is less strenuous than a traditional neuropsychological evaluation which makes it especially suitable for implementation in busy clinical care trajectories and for screening of vulnerable patients with a variety of neurological illnesses that commonly present with cognitive deficits. The downside of CNS VS being a brief screening is that its measurements are less comprehensive and that some subtests likely have a lower signal-to-noise ratio (C. Gualtieri & Johnson, 2006; S. J. M. Rijnen et al., 2018). Large-scale screening of patients, however, can give us better insight into the cognitive problems of different patient groups.

The test–retest reliability of individual measures resulting from CNS VS was shown to range between 0.31 and 0.87 (median: 0.68; test interval average 62 days) (C. Gualtieri & Johnson, 2006) or 0.18 and 0.88 (median: 0.47; test interval 3 and 9 months; Rijnen et al., 2018; Pearson’s r/Spearman’s ρ) in samples of healthy participants. Moreover, the concurrent validity of individual outcome measures was shown to range between 0.17 and 0.79 (Pearson’s *r*) in a sample comprising patients with various neuropsychiatric disorders and a small number of healthy participants. This is with the exception of the continuous performance tasks for which concurrent validity was very limited with a correlation between the correct responses of CNS VS Continuous Performance Task and the NES2 Continuous Performance Task of 0.04 (C. Gualtieri & Johnson, 2006). The CNS VS battery is shown to be able to detect significant decreases in test scores relative to healthy participants for various patient groups, including patients with neuro-(onco-)logical illnesses (e.g., Keine et al., 2019; Langensee et al., 2022; Papathanasiou et al., 2014; Rijnen, 2019; Rijnen, Butterbrod, et al., 2020).

Most individual CNS VS tests provide multiple scores, such as the number of correct responses, errors, and reaction times. As a result, the seven core tests in the CNS VS battery provide a total of 30 different independent test measures. This makes the interpretation of the cognitive profile difficult and laborious, especially as the screening is intended as brief and more global than regular neuropsychological assessment. To improve clinical interpretability, the developers of CNS VS provide 10 different “clinical domain” scores (excluding two composites: the Neurocognition Index and Composite memory). These domain scores are uniformly weighted linear combinations of individual test scores (sum scores), four of which being based on scores from multiple tests in the battery (CNS Vital Signs, n.d.). These domain scores are based on theory but are not (yet) empirically validated despite multiple attempts (Brooks et al., 2019; C. T. Gualtieri & Hervey, 2015) and have a low divergent validity (Plourde et al., 2018). Moreover, an explanation of how these domain scores were derived from theory is missing. This is concerning given that they are commonly used (e.g., Lanou et al., 2023; Lewis et al., 2023; Pilipenko et al., 2022) and that they should be validated in the same manner as latent variable models (McNeish & Wolf, 2020).

Finding empirical evidence for the unobserved latent constructs (the factor structure) underlying the measured test scores resulting from a CNT allows us to better understand the different constructs it measures and validate the use of composite scores for clinical practice. Two papers attempted to provide empirical evidence for the structure of the CNS VS test battery using exploratory or confirmatory factor analysis (CFA); one in a sample of youth with diverse neurological diseases (Brooks et al., 2019) and one in healthy adults (C. T. Gualtieri & Hervey, 2015). The factor structures in both investigations did not just differ from the “clinical domain” scores, but they also partly differed between the two samples. Both studies find three factors where two factors are interpreted as “(processing) speed” and “memory,” with several differences between the speed factors. A third factor was found in healthy adults, describing “attention,” while in the sample of youth with neurological disease, and the third factor described “inhibition.” This leads to the question of what is measured by CNS VS, and if the “clinical domains” are a valid representation of its structure. A further caveat is that both empirical studies assess only one group and, as such, cannot directly address whether the battery measures the same across different (diagnostic) populations, which is the problem of measurement invariance (Leitgöb et al., 2022).

Establishing measurement invariance is necessary for the valid use of composite scores, such as the clinical domain scores that are often automatically computed by computerized batteries, across various (diagnostic) groups. Measurement invariance can be divided into four increasingly strict levels: configural, metric, scalar, and residual. Configural invariance requires the same number of factors and approximately the same pattern of non-zero loadings between groups, indicating that the same constructs are measured. Metric invariance requires the same magnitude of loadings, allowing for testing the predictive relationship of a factor and some dependent variable. Scalar invariance requires approximately the same magnitude of intercepts, allowing for the comparison of latent means. Finally, residual invariance requires similar residuals between groups, indicating similar precision across groups (Chen, 2008; Hirschfeld & von Brachel, 2014). Unfortunately, measurement invariance regularly cannot be established (Wicherts, 2016).

In the current study, measurement invariance of CNS VS was tested across patients with three types of primary brain tumors (meningioma, low-grade glioma, and high-grade glioma) and healthy participants. Ideally, clinicians can be presented with easily interpretable test measures in clinical practice that have uniform interpretation for patients regardless of their diagnosis, which requires at least configural invariance between patient groups. Moreover, scores generally are normed relative to the performance of healthy participants to facilitate interpretation, thus requiring scalar invariance between all groups. Problems with cognition, however, are more common and more severe for patients with a primary brain tumor than for healthy participants, and neuropsychological functioning often is lower for patients with an isocitrate dehydrogenase (IDH) wild-type or high-grade glioma than those with an IDH-mutant or low-grade glioma (Noll et al., 2015; van Kessel et al., 2017, 2019). Moreover, the profile of cognitive profile is known to differ between patients and has been related to tumor grade (Butterbrod, 2021). This leads to the question if CNS VS measures the same constructs across these groups.

In this work, we performed exploratory factor analyses (EFA) on datasets consisting of 169 healthy Dutch individuals, 392 meningioma, 99 low-grade glioma, and 247 high-grade glioma patients separately; interpreted the factor structures; and tested model fit using CFA. Furthermore, we tested levels of measurement invariance across groups using multiple-group confirmatory factor analysis (MG-CFA) to be able to subsequently compare the latent means between the different groups (if scalar invariance was found) and to evaluate the use of the obtained factor structures for future work and use in clinical practice.

## Method

### Participants

Participants were meningioma and low- or high-grade glioma patients who were scheduled for surgery at the Elisabeth-TweeSteden hospital, Tilburg, the Netherlands, and underwent preoperative cognitive screening between 2010 and 2019. Patients were not included when they were under 18, had a progressive neurological disease, had a psychiatric or acute neurological disorder within the past 2 years, or had reduced testability for the neuropsychological assessment, for example, due to problems with vision, motor deficits such as paresis, and language deficits that complicated administration of the test battery. In addition, for comparison, data from healthy Dutch adults were collected using convenience sampling. Healthy participants were considered healthy if there was no past or present psychiatric or neurologic disorder, they had no other major medical illnesses in the past year before participation (e.g., cancer, myocard infarct), they were free of the use of any centrally acting psychotropic medication, and did not have a history of or current alcohol or drug abuse (Rijnen, Meskal et al., 2020).

The current samples are described (in part) in previous studies (Beele et al., 2024; Boelders et al., 2023, 2024; Butterbrod et al., 2019, 2020, 2021; De Baene, Rijnen, et al., 2019; De Baene, Rutten, et al., 2019; Lonkhuizen et al., 2019; Meskal et al., 2015; Rijnen, Butterbrod, et al., 2020; Rijnen, 2019; Rijnen, Kaya, et al., 2020; van der Linden et al., 2020; van Loenen et al., 2018).

### Design

During the screening using CNS VS, participants were accompanied by a well-trained technician (neuropsychologist or neuropsychologist in training) who provided instructions before starting each test. The test battery administered in our clinical practice included the seven core tests and took approximately 30–40 minutes to complete. The technician reported on the potential confounding of test scores caused by problems during administration (e.g., external disturbances). Test scores affected by confounding factors were excluded from the current study on a test-by-test basis. A description of the seven core tests used in this work can be found in Supplemental Table S1.

For all participants, a standardized interview was performed to obtain demographic variables (age, sex, and education). Education was recorded on the Dutch Verhage scale (Verhage, 1965). Data on race and ethnicity were not collected. Participants signed informed consent during this interview.

### Processing of Cognitive Measurements

#### Standardization of Raw Scores

We used 26 out of the 30 raw test scores resulting from the seven tests in CNS VS, leaving out the reaction times of the memory tasks. Reaction times of the memory tasks were left out as they make up a large number of variables while their interpretation is unclear and is in line with the work of Brooks and colleagues (2019). Test scores representing errors or reaction times were inverted such that a higher score represented a better performance. For all datasets (healthy participants and patients), test scores were converted to sociodemographically adjusted z-scores. This was done in two steps. First, test scores were corrected for the effects of age, sex, and education as found in the dataset of the same healthy participants using a multiple regression approach (Rijnen, Meskal et al. 2020). This was done as age and education are known to strongly affect test scores resulting in variance that is not informative of neuropsychological problems. Second, test scores of healthy participants were set to have a mean of zero and a standard deviation of one after which all patient groups were scaled relative to the healthy participants to facilitate interpretation.

#### Outliers

Test scores of the patient group were passed through a Winsor function to reduce the influential effect of univariate outliers on the analysis. The Winsor function sets the value of outliers after a certain percentile to the value at that percentile. This method was chosen because of its ability to maintain the information stored in most outliers while eliminating their influential effect on the analysis. The Winsor function was set to change only the most extreme outliers with a threshold of 3% on each side. The Winsor function was not applied to ends of the distributions with a ceiling or floor effect, precluding the Winsor function from increasing the ceiling or floor effects.

#### Missing Test Scores

Patients were removed from the analysis when three or more complete tests out of the seven tests in the test battery were missing. This threshold was chosen to ensure sufficient information for imputation. The remaining missing values were imputed using multiple imputation, estimating each test score from all the others as recommended by Zygmont and Smith (2014). Multiple imputation was chosen a priori, as missings in the screening are known to be missing at random. For example, test scores can be missing if patients do not understand or cannot execute the instruction of a (more complicated) task, which is reflected in poorer performance on other tasks. In addition, multiple imputation allows us to take the uncertainty of the imputations into account while performing the exploratory factor analysis and CFA. Details on imputation for the specific analyses follow below.

#### Multicollinearity

Test scores were evaluated for high multicollinearity using the variance inflation factor (VIF), where a VIF > 5 was interpreted as high multicollinearity. Test scores with high multicollinearity were combined (Paul, 2006). For consistency, tests with high multicollinearity in one sample were also combined in the other samples.

The prerequisite of mild multicollinearity necessary for the EFA was demonstrated using a Kaiser-Meyer-Olkin test between 0.5 and 1 and a significant (*p* < .005) Bartlett’s test of sphericity (Zygmont & Smith, 2014).

### Analysis

#### Exploratory Factor Analysis

To identify a factor structure for each group, four EFAs were performed. To determine the number of factors to maintain in the EFAs, we used traditional parallel analysis with principal component analysis as the extraction method and the mean as the reference value (Horn, 1965). Furthermore, we used the Empirical Kaiser Criterion and the Hull method implemented with the comparative fit index (CFI). This set of methods was based on recommendations by Auerswald and Moshagen (2019) given non-normally distributed data and were evaluated individually for each group.

The EFAs were performed using ordinary least squares (OLS) from the R package Psych (Revelle, 2017) (fa function with fm = “minres”) which aims to find the minimum residual solution (Harman & Jones, 1966). OLS was chosen as it is suitable for smaller samples while being robust when univariate and multivariate normality assumptions are violated (Zygmont & Smith, 2014).Oblimin rotation was used to further refine our factors as cognitive functions are highly dependent on one another, and oblimin rotation allows for correlations between latent factors (Browne, 2001). Missing test scores were imputed with multiple imputation with the R package Mifa (Nassiri, 2018) using the Multivariate Imputation by Chained Equations (MICE) algorithm with predictive mean matching as recommended by McNeish (2017).

Next, covariance matrices of the imputed datasets were combined using Rubin’s rules (Barnard & Rubin, 1999) after which the EFA was performed on the combined matrix. Factor interpretation was done over a series of meetings between the first author (S.B) and two neuropsychologists (E.B, K.G) who have extensive experience with both the patient groups and the CNS VS test battery. Interpretations were made over a series of meetings and revision rounds until a consensus was reached.

#### Confirmatory Factor Analysis

To determine the validity of the resulting factor structures, the fit of the resulting models was tested using CFA in the same groups where they were initially found. No separate validation sets were used due to the small sample sizes. Confirmatory models were defined as all non-zero factor loadings (loadings ≥|0.30|). As no validation set was used, no changes were made to the models based on residuals to prevent overfitting the models to the current sample. Models were considered to have a good fit when they had a root mean square error of approximation (RMSEA) < 0.06 and standardized root mean square residual (SRMR) < 0.06 (Hu & Bentler, 1999; Schreiber et al., 2006; Zygmont & Smith, 2014). If a good fitting model was not identified, the same confirmatory analyses were additionally performed while extracting different numbers of factors than done during the initial EFAs. This was done to prevent missing a good fitting model due to extracting too few or too many factors. The number of factors maintained was varied within the previously described metrics for factor extraction plus and minus one.

##### Measurement Invariance

To test if the found structure of CNS VS was invariant across different groups, measurement invariance between all—or a subset of groups—was tested using MG-CFA. To do so, the same patterns of non-zero factor loadings had to be found between EFA results, allowing for these results to be combined into one confirmatory model.

If patterns of non-zero loadings were the same between all—or a subset of—groups, configural invariance was tested by evaluating if the MG-CFA fit measures evaluated on these groups were good (Leitgöb et al., 2022). The MG-CFA was evaluated on a balanced sample comprising an equivalent number of randomly selected individuals from each group to ensure that the results were not biased by differences in sample size (Chen, 2007). Metric, scalar, and residual invariance were tested by comparing the change in CFI, RMSEA, and SRMR between models. We adhered to the stringent criteria as proposed by Chen (2007), where non-invariance for loadings (metric invariance) is indicated by a change in CFI ≥−.010 and RMSEA ≥−.015 and a change in SRMR ≥ -.030, and non-invariance for intercepts (scalar invariance) or residuals (residual invariance) is indicated by a change in CFI ≥−.010 and RMSEA ≥−.015 and a change in SRMR ≥−.0.10. If scalar invariance held, differences in latent means were tested by additionally restricting the means to be equal across groups for the scalar or strict model (depending on the level of invariance) and inspecting the change in model fit. A decreased model fit due to restricting the latent means was interpreted as evidence for unequal latent means across groups. If this was the case, the intercepts for the latent variables resulting from the scalar or strict model were reported.

Else, if patterns of non-zero loadings resulting from the EFA between all or a subset of groups were only *approximately* the same instead of being largely the same, measurement invariance was tested using EFA solutions resulting from a combined sample consisting of the groups with approximately the same patterns of non-zero loadings. This was done as sample sizes in this study for individual groups are relatively small (with as few as 99 samples) when compared to sample size recommendations such as 200 (Guilford, 1954) to 500 (Cattell, 2012) samples, or between 3:1 and 6:1 (Cattell, 2012) up to 20:1 (Hair et al., 1979) samples per variable. This small sample size may lead to sample-specific EFA solutions or poor model fit for the individual groups (Kyriazos, 2018; Taasoobshirazi & Wang, 2016).

The combined sample was created by randomly sampling an equivalent number of participants from each of the groups with approximately the same pattern of non-zero loadings. Multiple EFAs were performed on this combined sample where different numbers of factors were maintained. The resulting models were used to test measurement invariance between the groups that were part of the combined sample. The steps taken were the same as for the EFA and MG-CFA as previously described (i.e., determine the number of factors to maintain, perform EFAs, maintain loadings ≥|0.3,| and perform MG-CFAs). For both the EFAs and the MG-CFAs, a balanced sample was used to ensure that the results were not biased by differences in sample sizes (Chen, 2007). The number of factors maintained was again varied between the metrics for factor extraction plus and minus one to prevent missing an invariant model due to under- or over-extracting factors. If no configural variance was found, model fit was explored for the individual groups using CFAs. This was done to investigate if the fit for any group was particularly different.

The CFA and MG-CFA were performed using the R package Lavaan (Rosseel, 2012) with the maximum likelihood estimator with robust standard errors and a Satorra-Bentler scaled test statistic. Missing test scores were again imputed with multiple imputation using the MICE algorithm with predictive mean matching. The runMI function from the Semtools package was used to pool CFA parameter estimates, standard errors, and fit measures resulting from the different imputed datasets. Robust versions of the RMSEA (Brosseau-Liard et al., 2012), CFI (Brosseau-Liard & Savalei, 2014), and the Satorra-Bentler corrected version of the chi-square test statistic and SRMR (Satorra & Bentler, 2001) were used as they are robust to violations of the multivariate normality assumptions.

#### Factor Similarity

If configural invariant models could not be found, Tucker’s congruence coefficients were calculated between the initial EFA solutions of the individual samples instead, to aid the comparison of the different factor structures between groups. The Tucker’s congruence coefficient is the cosine angle between two vectors (representing the factors) (Tucker & Ledyard, 1951) and is commonly used to quantify the similarity between the factors resulting from an EFA solution (Lorenzo-Seva & ten Berge, 2006). A congruence coefficient between 0.85 and 0.94 was interpreted as fair similarity, and a value higher than 0.95 was interpreted as good similarity (Lorenzo-Seva & ten Berge, 2006).

## Results

### Sample Characteristics and Preprocessing

Data from 8 patients with a high-grade glioma, 12 patients with a meningioma, and 2 healthy participants were removed due to too many missing values. After these exclusions, data from 380 meningioma patients, 239 high-grade glioma patients, 99 low-grade glioma patients, and 167 healthy participants remained. Missing test scores comprised 2.94%, 0.11%, 3.71%, and 1.00% of the data for meningioma, low-grade glioma, high-grade glioma, and healthy participants, respectively. Sample characteristics of healthy participants and each patient group (after exclusions) can be found in Table 1. For a detailed description of the normative sample, we refer to Rijnen, Meskal et al. (2020).

**Table 1:** Sample Characteristics

Sample	Low-grade glioma	High-grade glioma	Meningioma	Healthy participants
Count	99	239	380	167
Age				
Mean (*SD*)	42.7 (13.4)	57.5 (12.4)	57.2 (12.0)	47.0 (15.1)
Min	19	18	23	20
Max	71	81	84	85
Education verhage (%)				
I	1.0%	0.4%	0.8%	0.0%
II	1.0%	29.8%	4.5%	0.0%
III	2.0%	4.6%	3.9%	0.0%
IV	16.2%	25.9%	28.4%	12.0%
V	34.3%	35.1%	28.6%	36.5%
VI	32.3%	23.8%	27.9%	36.0%
VII	13.1%	7.1%	5.8%	15.6%
Sex (% male)	56.6%	69.5%	27.4%	40.7%

*Note.* Sample characteristics of healthy participants and patient groups (after exclusions). Education was recorded on the Dutch Verhage scale (ranging from I to VII).

The correct responses of the continuous performance test were removed for further analysis as the number of correct responses and the omission errors on this task were the exact inverses of one another. High multicollinearity (VIF > 5) was found for the number of correct responses and errors in the shifting attention test. Therefore, we calculated a combined score of correct responses minus errors. All 24 remaining test scores had a VIF of at most 3.97. Descriptive statistics of the test results (count, mean, standard deviation, quantiles, skewness, kurtosis) after preprocessing are provided in Table 2. Moreover, this table describes the percentage of patients affected by the Winsor functioning, individually for each test score.

**Table 2:** Descriptives of the Neuropsychological Test Scores as Collected Using CNS Vital Signs.

Test measure	Count (out of 885)	Mean	Standard deviation	Minimum	25^th^ percentile	50^th^ percentile	75^th^ percentile	Maximum	Skewness	Kurtosis	Winsor (% of patients)
Verbal memory, initial correct hits	870	−0.36	1.30	−3.68	−1.14	−0.15	0.64	2.28	−0.71	−0.03	4.74
Verbal memory, initial correct passes	870	−0.38	1.52	−5.14	−1.30	−0.11	0.76	1.47	−1.41	1.53	4.74
Verbal memory, delayed correct hits	868	−0.34	1.18	−3.81	−1.13	−0.24	0.53	2.07	−0.38	−0.45	5.01
Verbal memory, delayed correct passes	868	−0.24	1.51	−5.27	−0.81	0.36	0.77	1.65	−1.62	2.41	5.01
Visual memory, initial correct hits	880	−0.40	1.37	−4.04	−1.13	−0.16	0.60	2.02	−0.73	0.10	3.48
Visual memory, initial correct passes	880	0.01	1.19	−4.02	−0.72	0.12	0.86	2.83	−0.39	−0.31	3.48
Visual memory, delayed correct hits	876	−0.22	1.40	−3.29	−1.07	−0.04	0.81	2.42	−0.40	−0.52	4.04
Visual memory, delayed correct passes	877	−0.24	1.39	−3.65	−1.12	−0.11	0.84	3.03	−0.49	−0.26	3.76
Symbol digit coding, correct hits	885	−0.75	1.30	−3.63	−1.62	−0.64	0.16	3.30	−0.23	−0.36	5.85
Symbol digit coding, errors	875	0.03	0.91	−5.08	−0.35	0.28	0.71	0.99	−1.67	3.21	4.32
Stroop simple, reaction time	885	−0.88	2.06	−7.69	−1.55	−0.19	0.49	1.67	−1.65	2.41	5.85
Stroop congruent, correct hits	845	−1.34	3.48	−15.39	−0.91	0.18	0.35	0.69	−2.70	7.00	7.94
Stroop congruent, reaction time	849	−1.23	2.18	−6.96	−2.43	−0.59	0.37	2.51	−0.94	0.19	7.38
Stroop congruent, commission errors	838	−0.51	1.76	−6.94	−1.06	0.12	0.70	1.33	−1.93	3.88	9.19
Stroop incongruent, correct hits	771	−1.13	4.17	−19.45	0.10	0.24	0.37	0.83	−3.38	11.01	18.52
Stroop incongruent, reaction time	883	−0.82	1.88	−6.49	−1.57	−0.34	0.47	1.90	−1.21	1.15	5.85
Stroop incongruent, commission errors	771	−0.30	1.55	−5.35	−1.15	0.43	0.70	1.28	−1.81	2.88	18.52
Shifting attention task, correct	851	−1.22	1.38	−4.37	−2.16	−1.02	−0.18	1.80	−0.48	−0.50	7.10
Shifting attention task, errors	851	−1.94	1.77	−6.63	−2.79	−1.36	−0.67	1.39	−1.09	0.40	7.10
Shifting attention task, reaction time	881	−0.26	1.24	−2.61	−1.18	−0.26	0.60	3.01	0.24	−0.21	5.85
Continuous performance test, omission errors	880	−1.33	3.81	−16.42	0.12	0.19	0.24	0.47	−2.83	7.46	3.48
Continuous performance test, commission errors	879	−0.27	1.57	−6.45	−0.74	0.39	0.62	1.53	−2.34	5.48	3.48
Continuous performance test, reaction time	884	−0.56	1.39	−4.30	−1.32	−0.36	0.45	1.83	−0.76	0.28	5.85
Finger-tapping test, average right	863	−0.63	1.35	−3.93	−1.47	−0.56	0.27	4.15	−0.24	0.07	5.99
Finger-tapping test, average left	862	−0.57	1.36	−3.99	−1.35	−0.43	0.40	4.27	−0.49	0.18	6.13

*Note.* Descriptive statistics of the test scores resulting from all healthy participants and patient groups together as used for the analysis (after preprocessing). Descriptive statistics for each individual group can be found as an online supplement to this study. Scores are scaled relative to healthy participants which were scaled to have a mean of zero and a standard deviation of one.

Visual inspection showed that the data were not normally distributed (several scores showed strong floor/ceiling effects). Kaiser-Meyer-Olkin scores were 0.82, 0.65, 0.77, and 0.67 for meningioma, low-grade glioma, high-grade glioma patients, and healthy participants, respectively. Bartlett’s test of sphericity was significant for all groups. The assumption of mild multicollinearity was met, and all datasets thus were suitable for the planned analyses.

### Number of Factors

For healthy individuals, Empirical Kaiser Criterion, the Hull method, and parallel analysis indicated 3, 7, and 5 factors as the optimal number to be extracted, respectively. For patients with a meningioma, these numbers were 3, 1, and 5 factors; for patients with a low-grade glioma, these were 3, 14, and 4; and for patients with a high-grade glioma, these were 3, 1, and 5 factors. Thus, Empirical Kaiser Criterion reported 3 factors across all samples, the Hull method ranged from 1 to 14 factors depending on the sample, and the parallel analysis test indicated 4 or 5 factors. Based on the factor retention rules, we argue that the optimal number of factors is between 3 and 5. Given the desire to compare factor structures across groups and the interpretability of the resulting factor solutions, we continued with 5 factors for each group.

### EFA and Factor Interpretation

#### Healthy Individuals

Table 3 describes the oblimin rotated five-factor solution of the EFA on the sample of healthy individuals. The total amount of variance explained by the exploratory model on the sample of healthy individuals was 34%. Based on our cutoff of 0.3 for factor loadings, 4 out of 24 variables did not load on any of the factors, and two variables loaded on two factors. Correlations between factors were small with one medium correlation of 0.35 between Factors I and IV (information-processing speed and motor speed).

**Table 3:** Exploratory Results for the Sample of Healthy Participants.

Test measure	Information-processing speed	General cognitive performance	Recognition of verbal material	Motor speed	Recognition of visual material	Communalities	Uniqueness
Verbal memory, initial correct hits	0.04	−0.01	**0.75**	0.04	0.03	0.58	0.42
Verbal memory, initial correct passes	0.12	0.25	0.04	−0.11	−0.1	0.08	0.92
Verbal memory, delayed correct hits	−0.02	0.01	**0.87**	0.02	0.02	0.77	0.23
Verbal memory, delayed correct passes	0.02	**0.34**	0.07	−0.14	−0.1	0.13	0.87
Visual memory, initial correct hits	−0.01	0.05	0.16	−0.14	**0.63**	0.52	0.48
Visual memory, initial correct passes	−0.06	**0.45**	0.00	−0.02	−0.18	0.25	0.75
Visual memory, delayed correct hits	−0.04	−0.12	0.00	0.05	**0.68**	0.50	0.50
Visual memory, delayed correct passes	−0.09	**0.57**	0.04	−0.03	−0.15	0.34	0.66
Symbol digit coding, correct hits	0.14	**0.44**	−0.03	0.21	0.07	0.35	0.65
Symbol digit coding, errors	−0.07	−0.04	−0.1	−0.09	−0.02	0.04	0.97
Stroop simple, reaction time	**0.59**	−0.19	0.08	0.08	−0.19	0.39	0.61
Stroop congruent, correct hits	0.13	0.12	**0.3**	−0.07	−0.07	0.11	0.89
Stroop congruent, reaction time	**0.84**	0.05	0.04	−0.01	−0.05	0.72	0.28
Stroop congruent, commission errors	−0.18	0.26	0	−0.04	−0.08	0.09	0.91
Stroop incongruent, correct hits	−0.01	0.06	−0.03	0.2	0.11	0.06	0.94
Stroop incongruent, reaction time	**0.60**	0.17	−0.07	0.07	0.14	0.52	0.48
Stroop incongruent, commission errors	−**0.44**	0.13	−0.11	**0.32**	0.00	0.20	0.80
Continuous performance test, omission errors	0.06	0.26	−0.06	−0.10	0.14	0.10	0.90
Continuous performance test, commission errors	−0.10	**0.43**	0.03	0.05	0.02	0.16	0.84
Continuous performance test, reaction time	**0.54**	−0.04	−0.13	0.15	0.10	0.39	0.61
Shifting attention task, reaction time	**0.39**	**0.33**	−0.04	0.13	0.12	0.40	0.60
Shifting attention task, correct minus errors	0.19	**0.55**	−0.05	0.06	0.05	0.43	0.57
Finger-tapping test, average left	0.01	−0.02	0.04	**0.81**	−0.06	0.72	0.28
Finger-tapping test, average right	0.05	0.03	0.05	**0.68**	0.04	0.49	0.51
Proportion Var	0.1	0.08	0.06	0.06	0.05		
Cumulative Var	0.1	0.17	0.23	0.3	0.34		
Factor correlations:							
Information-processing speed							
General cognitive performance	0.23						
Recognition of verbal material	0.06	−0.09					
Motor speed	0.35	0.21	0.06				
Recognition of visual material	0.03	0.00	0.21	−0.06			

*Note.* Results for the exploratory factor analysis on the sample consisting of healthy participants including the explained variance and correlations between factors. Loadings ≥|0.30| are presented in bold.

##### Interpretation

We interpret Factor I (Table 3) as found for the healthy individuals as describing “information-processing speed” (10% of the variance). This factor contains all reaction times. This factor additionally includes a negative loading for the commission errors of the Stroop incongruent condition, measuring inhibition of a dominant response. We argue that this may reflect a trade-off or strategy where the participant either presses quickly and risks a commission error or takes the time leading to a slower reaction time and fewer commission errors.

We interpret Factor II as describing “general cognitive performance” (8% of the variance). This factor includes the correct passes on both memory tasks (except for the initial correct passes of the verbal memory task). This factor additionally includes the correct minus errors and reaction time of the shifting attention task and the symbol digit coding task correct responses.

We interpret Factor III as describing “recognition of verbal material” (6% of the variance) as it includes the correct hits on the initial and delayed condition of the verbal memory recognition task, that is, correctly identifying target words among distractor words. This factor additionally includes, to a lesser extent, the correct responses of the congruent Stroop task, measuring color-word matching (with a loading of 0.30 versus loadings of over 0.75 and 0.87 for the initial and delayed correct hits, respectively).

We interpret Factor IV as describing “motor speed” (6% of the variance). This factor includes the left and right conditions of the finger-tapping task which are simple tests of motor control. Although the factor loading of the commission errors of the incongruent Stroop task was 0.32 on this factor, it loaded stronger on the information-processing speed factor (−0.44).

We interpret Factor V as describing “recognition of visual material” (5% of the variance) as it only includes the correct hits on the initial and delayed condition of the visual memory recognition task, that is, correctly remembering target figures among distractor figures.

#### Meningioma Patients

Table 4 describes the EFA solution for the first sample of meningioma patients. The total amount of variance explained was 46%. All variables loaded on at least one factor and two variables loaded on two factors. Correlations between factors were generally small with one medium correlation of 0.46 for Factors I (attention) and V (motor speed) and one medium negative correlation between Factors II (memory correct passes/strategy) and III (recognition of visual and verbal material).

**Table 4:** Exploratory Results for Patients With a Meningioma.

Test measure	Attention	Memory correct passes/strategy	Recognition of visual and verbal material	Inhibition	Motor speed	Communalities	Uniqueness
Verbal memory, initial correct hits	0.03	0.00	**0.80**	0.06	−0.06	0.64	0.36
Verbal memory, initial correct passes	−0.05	**0.49**	−0.04	0.16	0.11	0.32	0.68
Verbal memory, delayed correct hits	−0.08	0.05	**0.71**	−0.10	0.04	0.46	0.54
Verbal memory, delayed correct passes	0.05	**0.53**	−0.04	**0.32**	0.05	0.52	0.48
Visual memory, initial correct hits	0.16	−0.24	**0.5**	0.07	0.02	0.46	0.54
Visual memory, initial correct passes	−0.04	**0.73**	0.01	−0.11	−0.02	0.50	0.50
Visual memory, delayed correct hits	−0.02	−**0.3**	**0.42**	0.07	0.12	0.40	0.60
Visual memory, delayed correct passes	0.09	**0.63**	−0.11	0.03	−0.07	0.49	0.51
Symbol digit coding, correct hits	**0.37**	0.14	0.10	0.13	0.28	0.45	0.55
Symbol digit coding, errors	0.04	−0.09	−0.10	**0.30**	0.09	0.11	0.89
Stroop simple, reaction time	**0.58**	−0.07	0.05	−0.11	0.17	0.44	0.56
Stroop congruent, correct hits	**0.34**	−0.09	0.05	**0.33**	−0.03	0.24	0.76
Stroop congruent, reaction time	**0.84**	−0.04	−0.07	0.00	0.00	0.66	0.34
Stroop congruent, commission errors	−0.07	0.03	0.06	**0.62**	0.10	0.41	0.59
Stroop incongruent, correct hits	**0.68**	0.03	−0.01	0.22	−0.07	0.55	0.45
Stroop incongruent, reaction time	**0.85**	0.01	0.04	−0.08	−0.01	0.71	0.29
Stroop incongruent, commission errors	0.21	0.04	−0.01	**0.58**	0.08	0.52	0.48
Continuous performance test, omission errors	**0.30**	0.11	0.17	0.09	0.08	0.21	0.79
Continuous performance test, commission errors	0.03	0.20	0.08	**0.50**	0.05	0.36	0.64
Continuous performance test, reaction time	**0.53**	0.00	0.05	−0.17	0.16	0.38	0.62
Shifting attention task, reaction time	**0.34**	0.13	0.05	−**0.42**	0.11	0.27	0.73
Shifting attention task, correct minus errors	**0.66**	0.11	0.04	0.12	0.05	0.57	0.43
Finger-tapping test, average left	0.04	−0.04	0.00	0.09	**0.77**	0.67	0.33
Finger-tapping test, average right	−0.02	0.01	−0.01	−0.03	**0.90**	0.77	0.23
Proportion Var	0.16	0.08	0.08	0.07	0.08		
Cumulative Var	0.16	0.23	0.31	0.39	0.46		
Factor correlations:							
Attention							
Memory correct passes	0.15						
Recognition of visual and verbal material	0.22	−0.33					
Inhibition	0.22	0.23	0.08				
Motor speed	0.46	0.03	0.23	0.18			

*Note.* Results for the exploratory factor analysis for the sample consisting of patients with a meningioma including the explained variance and correlations between factors. Loadings ≥|0.30| are presented in bold.

##### Interpretation

We interpret Factor I (Table 4) as found for the meningioma patients as describing “attention” (16% of the variance). The factor includes all reaction time scores, the number of correct responses for the symbol digit coding task, the congruent Stroop task, the incongruent Stroop task, the omission errors and reaction time of the continuous performance task, and the shifting attention task.

We interpret Factor II as describing “memory correct passes/strategy” (8% of variance). This factor includes the correct passes of all memory tasks, that is, correctly identifying distractors and not responding to these while under time pressure. It additionally loads negatively on the initial and delayed correct hits of the visual memory task, indicating a strategy component for this memory task. A participant may have a better memory, resulting in more correct hits and more correct passes. On the other hand, participants may use a strategy (e.g., not pressing when uncertain) or may have problems responding, resulting in more correct passes while also reducing the number of correct hits. The same argument can be made for the memory correct passes factors of the groups below.

We interpret Factor III as describing “recognition of visual and verbal material” (8% of variance). This factor only includes the correct hits of all memory tasks, that is, correctly identifying target words among distractor words.

We interpret Factor IV as describing “inhibition” (7% of variance) as this factor includes the commission errors and correct hits of the congruent Stroop task, the commission errors of the incongruent Stroop task, the commission errors of the continuous performance task, the correct passes of the verbal memory recognition task, and the errors of the symbol digit coding task. This factor additionally loads negatively on the reaction time of the shifting attention task, which may indicate a trade-off between speed and (commission) errors.

We interpret Factor V as describing “motor speed” (8% of the variance). This factor only includes the finger-tapping tests.

#### Low-Grade Glioma Patients

Table 5 describes the EFA solution for the complete sample of low-grade glioma patients. The total amount of variance explained was 47%. All variables except one loaded on at least one factor except for the commission errors of the continuous performance test. Six variables loaded on multiple factors. Correlations between factors were small with a maximum correlation of 0.43 between Factors I and II (psychomotor speed and attention). It is important to note that these results were obtained using a relatively small sample (*N* = 99), potentially leading to sample-specific results.

**Table 5:** Exploratory Results for Patients With a Low-Grade Glioma.

Test measure	Attention	Psychomotor speed	Inhibition	Recognition of visual and verbal material	Memory correct passes	Communalities	Uniqueness
Verbal memory, initial correct hits	−0.18	0.14	−0.02	**0.65**	0.15	0.44	0.56
Verbal memory, initial correct passes	−0.02	0.12	0.13	−0.06	**0.63**	0.46	0.54
Verbal memory, delayed correct hits	−0.08	−0.12	0.05	**0.61**	0.16	0.38	0.62
Verbal memory, delayed correct passes	0.15	0.06	0.00	−0.09	**0.71**	0.63	0.37
Visual memory, initial correct hits	0.09	0.05	0.00	**0.71**	−0.2	0.59	0.41
Visual memory, initial correct passes	0.22	0.27	0.01	−0.06	**0.33**	0.35	0.65
Visual memory, delayed correct hits	0.09	−0.18	−0.06	**0.65**	−0.14	0.49	0.51
Visual memory, delayed correct passes	0.30	0.06	−0.07	0.01	**0.58**	0.53	0.47
Symbol digit coding, correct hits	0.27	**0.38**	0.16	0.14	0.19	0.47	0.53
Symbol digit coding, errors	0.01	−**0.38**	0.12	−0.15	0.12	0.18	0.82
Stroop simple, reaction time	**0.50**	0.19	0.14	0.13	−0.26	0.46	0.54
Stroop congruent, correct hits	**0.31**	0.04	**0.39**	0.04	0.04	0.34	0.66
Stroop congruent, reaction time	**0.85**	−0.05	−0.06	−0.05	0.12	0.73	0.27
Stroop congruent, commission errors	−0.11	−0.05	**0.76**	−0.07	−0.02	0.53	0.47
Stroop incongruent, correct hits	**0.43**	−0.14	**0.52**	0.05	0.14	0.56	0.44
Stroop incongruent, reaction time	**0.75**	0.13	−0.04	0.05	0.16	0.76	0.24
Stroop incongruent, commission errors	−0.11	−0.07	**0.73**	0.02	0.03	0.47	0.53
Continuous performance test, omission errors	0.09	**0.35**	**0.43**	0.01	−0.16	0.38	0.62
Continuous performance test, commission errors	−0.06	0.12	0.15	0.08	0.21	0.08	0.92
Continuous performance test, reaction time	**0.50**	0.24	0.10	−0.12	−**0.36**	0.47	0.53
Shifting attention task, reaction time	0.02	**0.79**	-0.13	−0.11	−0.02	0.67	0.33
Shifting attention task, correct minus errors	0.00	**0.72**	0.02	0.05	0.20	0.59	0.41
Finger-tapping test, average left	0.15	**0.31**	**0.32**	0.14	0.02	0.34	0.66
Finger-tapping test, average right	0.14	**0.30**	**0.32**	0.20	−0.06	0.34	0.66
Proportion	0.12	0.10	0.09	0.08	0.08		
Cumulative	0.12	0.21	0.30	0.39	0.47		
Factor correlations:							
Attention							
Psychomotor speed	0.43						
Inhibition	0.21	0.06					
Recognition of visual and verbal material	0.03	0.01	0.13				
Memory correct passes	0.23	0.14	0.02	−0.12			

*Note.* Results for the exploratory factor analysis for the sample consisting of patients with a low-grade glioma including the explained variance and correlations between factors. Loadings ≥|0.30| are presented in bold.

##### Interpretation

We interpret Factor I (Table 5) as found for the low-grade glioma patients as describing “attention” (12% of the variance). This factor includes all reaction times except the reaction time on the shifting attention task. This factor additionally includes the correct responses of the congruent and incongruent Stroop task. Note that these last two scores load stronger on Factor III (Inhibition).

We interpret Factor II as describing “psychomotor speed” (10% of variance) as this factor includes both finger-tapping tasks. It also includes the reaction time and performance of the shifting attention task and the correct and errors of the symbol digit coding task. These loadings can be explained by the difficult response options for these tasks (e.g., the targets that should be pushed on the keyboard switch and need to be continuously updated with each item, requiring both cognitive flexibility and switching of efferent motor commands). Last, this factor contains the omission errors of the continuous performance task which we are unable to interpret and which loads stronger on Factor III (Inhibition)

We interpret Factor III as describing “inhibition” (9% of the variance). This factor includes the correct and commission errors of both Stroop tasks, the omission errors of the continuous performance task, and to a lesser extent, both finger-tapping tasks which also load on Factor II (psychomotor speed).

We interpret Factor IV as describing “recognition of visual and verbal material” (8% of variance) as this factor only includes the correct hits of all memory tasks, that is, correctly identifying target words among distractor words.

We interpret Factor V as describing “memory correct passes” (8% of variance). This factor includes the correct passes of all memory tasks. This factor additionally includes, to a lesser extent, negatively the reaction time on the continuous performance task. We are unable to interpret this last loading which loads stronger on Factor I (attention).

#### High-Grade Glioma Patients

Table 6 describes the EFA solution for the first sample of high-grade glioma patients. The total amount of variance explained was 44%. Two variables did not load on any factor, and one variable loaded on two factors. Correlations between factors were small with one medium correlation of 0.42 between Factors I and V (complex attention and motor speed).

**Table 6:** Exploratory Results for Patients With a High-Grade Glioma.

Test measure	Attention	Memory correct passes/Strategy	Inhibition	Recognition of verbal material	Motor speed	Communalities	Uniqueness
Verbal memory, initial correct hits	0.11	0.04	−0.06	**0.69**	0.03	0.51	0.49
Verbal memory, initial correct passes	0.19	**0.42**	0.03	−0.17	−0.03	0.27	0.73
Verbal memory, delayed correct hits	−0.09	−0.01	0.03	**0.87**	−0.01	0.73	0.28
Verbal memory, delayed correct passes	0.27	**0.33**	0.05	−0.22	−0.05	0.26	0.74
Visual memory, initial correct hits	**0.45**	−**0.49**	−0.07	0.18	0.08	0.56	0.44
Visual memory, initial correct passes	−0.03	**0.69**	−0.02	0.13	0.06	0.46	0.54
Visual memory, delayed correct hits	0.22	−**0.56**	−0.03	0.20	−0.01	0.46	0.54
Visual memory, delayed correct passes	0.22	**0.7**	0.02	0.01	0.06	0.59	0.41
Symbol digit coding, correct hits	**0.40**	−0.01	0.27	0.21	−0.01	0.39	0.61
Symbol digit coding, errors	0.02	−0.18	0.22	0.03	0.04	0.08	0.92
Stroop simple, reaction time	**0.61**	0.12	−0.11	0.00	0.18	0.50	0.50
Stroop congruent, correct hits	0.12	−0.09	**0.50**	0.07	0.1	0.33	0.67
Stroop congruent, reaction time	**0.65**	0.02	−0.01	0.02	0.11	0.52	0.48
Stroop congruent, commission errors	−0.1	−0.09	**0.54**	−0.15	0.03	0.26	0.74
Stroop incongruent, correct hits	**0.59**	0.09	0.26	0.07	0.02	0.55	0.46
Stroop incongruent, reaction time	**0.88**	−0.02	0.02	−0.03	−0.01	0.77	0.23
Stroop incongruent, commission errors	0.02	0.05	**0.75**	0.02	0.00	0.58	0.42
Continuous performance test, omission errors	**0.37**	0.01	0.11	0.01	0.17	0.26	0.74
Continuous performance test, commission errors	−0.01	0.09	**0.39**	−0.08	0.13	0.20	0.81
Continuous performance test, reaction time	**0.71**	−0.05	−0.04	−0.06	0.07	0.52	0.49
Shifting attention task, reaction time	0.22	0.05	−0.05	0.22	−0.16	0.10	0.90
Shifting attention task, correct minus errors	**0.49**	0.17	0.27	0.11	−0.09	0.45	0.56
Finger-tapping test, average left	0.00	−0.03	0.13	−0.03	**0.65**	0.43	0.57
Finger-tapping test, average right	0.00	0.03	−0.03	0.01	**0.92**	0.94	0.06
Proportion Var	0.16	0.08	0.07	0.07	0.06		
Cumulative Var	0.16	0.24	0.31	0.38	0.44		
Factor correlations:							
Attention							
Memory correct passes	0.07						
Inhibition	0.27	0.23					
Recognition of verbal material	0.27	−0.26	0.05				
Motor speed	0.42	0.10	0.10	0.10			

*Note.* Results for the exploratory factor analysis for the sample consisting of patients with a high-grade glioma including the explained variance and correlations between factors. Loadings ≥|0.30| are presented in bold.

##### Interpretation

We interpret Factor I (Table 6) as found for the low-grade glioma patients as describing “attention” (16% of variance). This factor includes all reaction time scores except for the reaction time of the shifting attention task. This factor additionally includes the correct responses (i.e., the accuracy) of the incongruent Stroop task, the shifting attention task, and the symbol digit coding test, all of which have a flexibility component. Last, this factor includes the correct hits of the visual memory tasks, although this score also loads stronger on Factor II (memory correct passes).

We interpret Factor II as describing “memory correct passes/strategy (8% of variance). This factor includes the correct passes of all memory tasks. It additionally includes the inverse of the initial and delayed correct hits of the visual memory task, indicating a strategy component for this memory task.

We interpret Factor III as describing “inhibition” (7% of the variance). This factor includes the commission errors of both Stroop tasks, the correct hits of the congruent Stroop task, and the commission errors of the continuous performance task. This task additionally includes the correct hits of the symbol digit coding task which we are unable to explain and which loads stronger on Factor I (attention)

We interpret Factor IV as describing “recognition of verbal material” (7% of variance). This factor only includes the verbal memory initial and delayed correct hits, that is, correctly identifying target words among distractor words.

We interpret Factor V as describing “motor speed” (6% of variance). This factor only includes the left and right finger-tapping tests.

### Confirmatory Factor Analysis

To determine the validity of the factor structures described above, the fit measures for four CFA models based on the four EFA solutions previously described were inspected and are presented in Supplemental Table S2. Results show that none of the resulting models had a good fit.

Therefore, the same analysis was also performed while maintaining between two and six factors, resulting in a total of 20 different models. These results are also presented in Supplemental Table S2 and did not indicate good model fit for any of the patient groups regardless of the number of factors maintained. For healthy participants, the RMSEA indicated a good model fit while maintaining four to six factors, but not while maintaining two or three factors. The SRMR, however, did not show good model fit for healthy participants regardless of the number of factors maintained. Therefore, we conclude that no models with a good fit could be found for individual groups using the current modeling approach.

### Measurement Invariance

To test measurement invariance, the same patterns of non-zero factor loadings resulting from the EFAs are required between all—or a subset of groups. The factor structures resulting from the EFA for healthy participants, however, differed from patient groups with different factor interpretations for most factors. Factor structures among patient groups were more similar, with approximately the same factor interpretations. Despite the similarities in interpretation among patient groups, results showed between-group differences in non-zero loadings for almost every pair of factors, with up to six loadings differing (between the inhibition factors of meningioma and low-grade glioma patients). Therefore, we did not continue with the planned tests of measurement invariance using the models resulting from the individual groups or a subset thereof.

As the patterns of non-zero loadings for the patient groups were *approximately* the same, measurement invariance was tested using EFA solutions resulting from a combined sample consisting of 99 participants from each patient group. For the combined sample, Bartlett’s test of sphericity was significant and the Kaiser-Meyer-Olkin score was 0.81 indicating that was sample was suitable for the planned analysis. Parallel analysis, the Empirical Kaiser Criterion, and the Hull method indicated 4, 3, and 1 as the optimal number to be extracted respectively. Therefore, measurement invariance was tested for models with one up to five factors.

Supplemental Table S3 shows the fit measures of the MG-CFAs (based on the EFA solutions resulting from the combined sample, not shown) as evaluated on all patients (one MG-CFA per number of factors maintained). None of the MG-CFAs indicated good model fit; therefore, we conclude that no configural invariant model could be identified using the current modeling approach regardless of the number of factors maintained. Supplemental Table S3 further shows the fit of the same confirmatory models when applied to the individual groups. None of the individual patient groups had a notably different fit according to these CFAs. This indicates that the lack of good model fit of the MG-CFA is not due to any specific sample. As a model with good fit is a prerequisite for configural invariance, we did not continue the analysis of measurement invariance. As scalar invariance is required to compare latent means, we also did not continue with the planned comparison of latent means.

As no model with configural invariance could be identified, Tucker’s congruence coefficients between each pair of factors resulting from the initial EFAs on the individual groups were calculated instead (Supplemental Table S4). Comparing factors for patients with low-grade glioma and patients with meningioma, we found that one factor had fair similarity and that no factors had good similarity. For patients with high-grade glioma and patients with a meningioma, we found that all five factors had fair similarity while no factors had good similarity. Between patients with high-grade and low-grade glioma we found that one factor had fair similarity and no factors had good similarity. Finally, no factors showed fair or good similarity between healthy participants and any of the patient groups (for which factors also differed in interpretation).

## Discussion

In this work, we performed four EFAs on the test scores of the CNS VS computerized neuropsychological screening in healthy participants and three samples of patients with primary brain tumors, evaluated model fit, and tested measurement invariance between groups. Results showed several differences in factor interpretation, especially when comparing healthy participants to patient groups. Factor structures among patient groups had approximately the same factor interpretation but differed in non-zero loadings for almost every pair of factors. No good fitting model could be found given the current modeling approach regardless of the group(s) used or the number of factors maintained. Therefore, configural invariance between groups could not be established.

### Implications for the Use of Composite Scores Resulting From CNS VS

Given that no model with good fit could be identified, regardless of the group(s) and the number of factors maintained, it is likely that the structure of CNS VS cannot be adequately captured using linear components (cf. C. T. Gualtieri & Hervey, 2015). This, in turn, raises concerns about the validity of using (weighted) sum scores of the metrics resulting from CNS VS, including the “clinical domains” that are provided to aid intuitive clinical interpretation. We therefore recommend researchers and clinicians using CNS VS to use scores resulting from the individual tests, rather than composite (e.g., domain) scores consisting of scores from multiple tests in the battery.

Furthermore, due to the lack of a model with good fit, configural invariance across groups could not be established. This is in line with previous research that performed evaluations of measurement invariance in a variety of (non) CNT batteries. Mixed results have been found when comparing patient groups to healthy participants or patients without cognitive impairments (Haring et al., 2015; Ma et al., 2021; Yang et al., 2022). Furthermore, measurement invariance did not hold for half of the studies reviewed by Wicherts (2016) that compared IQ test batteries across different subgroups. Therefore, we recommend users of neuropsychological test batteries to consider measurement invariance in addition to other validity measures before comparing sum scores between groups.

There can be multiple causes why a good fitting model could not be found using EFA. First, CNS VS is a brief neuropsychological screening with some tests likely having a lower signal-to-noise ratio when compared to more extensive tests, potentially complicating factor extraction (Watkins, 2018) and reducing fit measures (Niemand & Mai, 2018). Second, data were collected as part of a busy clinical care trajectory which likely contributes to the amount of noise in the data. Third, the incidence of primary brain tumors is relatively low, causing the sample size to be low despite being large for its kind. Fourth, several measures resulting from CNS VS are not normally distributed and/or suffer from floor or ceiling effects potentially complicating factor extraction (Watkins, 2018) and negatively affecting fit measures (Ainur et al., 2017; Ory & Mokhtarian, 2010). These effects are present for several test variables including the omission and commission errors of the continuous performance test and the correct hits of the congruent Stroop task. As a post hoc analysis excluding these tasks with strong floor/ceiling effects did not result in good model fit (results not shown), it is less likely that these effects were the main driver of the poor fit. Fifth, a more general explanation may be that variables resulting from CNTs, as from traditional cognitive tests (Agelink van Rentergem et al., 2020), may have more complex, non-linear interrelationships that cannot be sufficiently captured using linear factor analysis. This means that other representations of the test scores or other modeling approaches may result in better fitting models.

### Similarities and Differences in the Factor Structures Found in This Study

Comparison of factor structures between patient groups on the one hand and healthy participants on the other showed both differences and similarities in interpretation. First, information-processing speed for healthy participants and attention in patient groups were largely comparable (congruence between 0.76 and 0.80). However, the attention factor in patients contained accuracy scores in addition to reaction times, whereas it did not for healthy individuals. The inclusion of accuracy scores jointly with reaction time scores in one factor among patients could be due to a joint attention-processing speed deficit as a result of brain damage (Lezak et al., 2004; Mathias & Wheaton, 2007), resulting in stronger linkage of these two components in a factor than in healthy controls. Alternatively, it could be that mental slowness, one of the most common deficits in brain damage, leaves patients unable to timely process and use relevant information, resulting in more errors. Second, an Inhibition factor was found only for patient groups. This likely is explained by problems with inhibition being common in patients with brain lesions (Conway & Fthenaki, 2003; Picton et al., 2006). Problems with inhibition of a response may cause them to respond on stimuli where they should not, leading to a factor comprising most of the (commission) errors. Last, memory tasks were divided into visual memory recognition and verbal memory recognition for healthy participants, and into memory recognition and memory correct passes for patients. This finding for patient groups differs from most neuropsychological literature where memory is separated into visual and verbal memory or short-term and long-term memory (Kraemer et al., 2009; Lezak et al., 2004). This finding may be explained by a more general impairment in memory encoding for patient groups. Such an impairment affects both visual and verbal memory, and long- and short-term memory. This, in turn, likely causes patients to rely on a strategy for these tasks where they either indicate that they recognized a stimulus in case of doubt, or do not indicate that they recognize the stimulus.

Factor structures across patient groups also showed both similarities and differences. Patient groups always showed a factor interpreted as attention (congruence between 0.84 and 0.93) that captured the most variance, indicating that deficits in attention play an important role in their test performance. Moreover, patient groups always had two similar memory-related factors (congruence between 0.70 and 0.92). Last, patient groups always had an Inhibition factor (congruence between 0.74 and 0.86). However, for patients with a low-grade glioma, psychomotor speed was found instead of motor speed, measuring the integration between cognitive and motor skills. Moreover, memory correct passes did not contain a strategy component for patients with low-grade glioma, potentially being explained by patients with a low-grade glioma (known to have less severe cognitive impairments) being less reliant on strategies. Last, there were many differences in non-zero loadings between patient groups that did not change our interpretation. It is important to note that some differences in factor structure may be due to the relatively small sample-to-variable ratios (with as few as 99 participants), possibly leading to sample-specific solutions. Moreover, it is important to consider that correlations of skewed variables may have been over- or under-estimated (Bishara & Hittner, 2015), potentially affecting the EFA results (Watkins, 2018).

### Relating Factor Structures Found in This Study to Previous Studies

Comparing our results in the patient groups to results by Brooks and colleagues (Brooks et al., 2019) who performed an EFA in a sample of youth with diverse neurological diagnoses, we find that the structures were fairly similar, despite Brooks and colleagues only finding three factors. The memory factor by Brooks and colleagues is comparable to our recognition of visual and verbal material factors, indicating that CNS VS captures adequate recognition of (quick) responses to the target material across diagnoses. The Inhibition factor by Brooks and colleagues is comparable to the combination of our memory correct passes/strategy factors and our inhibition factors. This suggests that CNS VS may be sensitive to inhibition in different cognitive domains across diagnoses. Finally, the speed factor by Brooks and colleagues is comparable to the combination of our attention and (psycho-)motor speed factor. The rough similarities between the factor structures of patient groups in our work and the work by Brooks and colleagues suggest that factor structures in other patient groups may also be somewhat similar while likely not being invariant.

The CFA by C. T. Gualtieri and Hervey (2015) on healthy individuals also resulted in three factors. Overall, their Memory factor is like the recognition of visual and recognition of verbal material factors as found in our healthy sample, with the addition of the memory correct passes that we found in our General cognitive performance factor. Test scores in their processing speed or attention factor are either included in our information-processing speed factor or our general cognitive performance factor.

### Comparing the Factor Structures to the CNS VS “Clinical Domain” Scores

CNS VS provides 10 different, but partly overlapping, domain scores based on neuropsychological theory (CNS Vital Signs, n.d.). Our empirical approach, resulting in a five-factor solution, offered little support for the large number of domain scores. Both our results and previous studies indicate that it is difficult to distinguish between reaction time, processing speed, executive function, and simple attention using CNS VS, as suggested by the domain scores. At the same time, some support for some of the CNS VS domain scores was found. First, both the domain scores and our results distinguished aspects of memory and motor speed from all other functions. Furthermore, the variables in the CNS VS Reaction time and Executive function domains were part of the attention domains found in our patient groups. The discrepancy between the factors found using EFA and clinical domains may, in part, be explained by the difference in methodology. The EFAs aim to find distinct sources of common variance present in multiple variables. This causes the factor analysis to ignore some smaller sources of variance or combine overlapping sources of variance. This makes test measures less prone to be part of multiple factors, as is the case with several CNS VS domains. Furthermore, the standardized scores we used for our tests were computed using a different approach than CNS VS.

Similar to the findings of C. T. Gualtieri and Hervey (2015), our study leads us to conclude that CNS VS cannot be used to make thorough distinctions between cognitive domains. Finding few factors that mostly describe broad cognitive domains is in line with results for other brief (20–30 minutes) neuropsychological tests such as the Computer-Administered Neuropsychological Screen for Mild Cognitive Impairment (CANS-MCI) (Memória et al., 2014; Tornatore et al., 2005) and the (computerized) Cognitive Stability Index (CSI) (Erlanger et al., 2002). A notable exception from this is a recent meta-factor analysis of the Repeatable Battery for the Assessment of Neuropsychological Status (RBANS) (Goette, 2020). These results differ from extensive tests such as the Wechsler Adult Intelligence Scale—Fourth edition (WAIS-IV) which measure more detailed (intelligence) functions (Benson et al., 2010; Canivez & Watkins, 2010).

It is important to note that the current study does not rule out that models with good model fit and measurement invariance can be found using different modeling approaches, nor do our results directly address the validity of CNS VS’ individual tests. Moreover, the current study does not invalidate previous studies utilizing composite or “clinical domain” scores resulting from CNS VS. However, caution is warranted when interpreting results based on such scores. While they likely provide some indication of cognitive (dys)function, they may not linearly relate to, nor precisely describe the specific functions they are assumed to measure. Moreover, such scores may measure different functions in different (diagnostic) samples. Last, some caution is warranted when interpreting results that aim to distinguish between reaction time, processing speed, executive function, and simple attention, since these specific cognitive functions have not been identified in the current and previous factor analyses (Brooks et al., 2019; C. T. Gualtieri & Hervey, 2015)

### Suggestions for Future Work

As brief neuropsychological screenings are becoming increasingly popular instruments, including in clinical trials, more research into the factor structures of these batteries is needed. This is especially the case for computerized batteries as studies regarding their validity are lacking (De Roeck et al., 2019; Zygouris & Tsolaki, 2015). This is further emphasized by the concerning results for CNS VS with respect to the use of composite scores both within a specific group and in comparisons between multiple groups, as found in the current study.

Future work should consider excluding variables that have strong floor/ceiling effects and can consider modeling general intelligence or processing speed using a bi-factor or higher-order model (Beaujean, 2015). In addition, bi-factor models can be used to model residual correlations between outcome types or scores resulting from the same test. Besides aiming to improve model fit, future work can develop models based on established theory such as the Cattell–Horn–Carroll abilities (Schneider & McGrew, 2018) which may result in a model with a better fit or can use non-linear factor analysis. Alternatively, computational models of cognitive processes, such as those by Agelink van Rentergem and colleagues (2021), can be considered. For example, drift-diffusion models can model tasks where the participant receives conflicting stimuli (Ratcliff & McKoon, 2008; White et al., 2018). To apply these models, however, more detailed measures such as the response times for each stimulus are needed which are not provided by CNS VS. Finally, future research should continue to study the reliability and validity of individual tests in CNS VS for healthy participants and different patient populations and could study other sources of non-invariance such as tumor location.

## Conclusion

In this work, we explored the factor structures of the CNS VS neuropsychological test battery in patients with a meningioma, low-grade glioma, high-grade glioma, and in healthy participants, tested model fit, and investigated measurement invariance. Results showed several differences in factor interpretation between healthy participants and patient groups. Factor structures among patient groups were more similar when compared to the differences between patients and healthy participants, but differed in non-zero loadings for almost every pair of factors. Factor structures largely did not support the “clinical domains” proposed by CNS VS. No good fitting model could be found using the current modeling approach, raising concerns about the validity of composite (including “clinical domain”) scores resulting from CNS VS. Therefore, we recommend both clinicians and researchers to use test scores resulting from individual tests instead of combining scores resulting from multiple tests within the battery. Configural invariance could not be established due to the inability to identify a model with good fit. Given that measurement invariance often is not found in neuropsychological test batteries, we recommend users be mindful of measurement invariance in addition to other validity measures when comparing sum scores between groups.

## Supplemental Material

sj-docx-1-asm-10.1177_10731911241289987 – Supplemental material for Factor Structure and Validity of Composite Scores Resulting From a Computerized Cognitive Test Battery in Healthy Adults and Patients With Primary Brain Tumors Supplemental material, sj-docx-1-asm-10.1177_10731911241289987 for Factor Structure and Validity of Composite Scores Resulting From a Computerized Cognitive Test Battery in Healthy Adults and Patients With Primary Brain Tumors by S.M. Boelders, E. Butterbrod, L.V.D.E. Vogelsmeier, M.M. Sitskoorn, L.L. Ong and K. Gehring in Assessment

sj-docx-2-asm-10.1177_10731911241289987 – Supplemental material for Factor Structure and Validity of Composite Scores Resulting From a Computerized Cognitive Test Battery in Healthy Adults and Patients With Primary Brain Tumors Supplemental material, sj-docx-2-asm-10.1177_10731911241289987 for Factor Structure and Validity of Composite Scores Resulting From a Computerized Cognitive Test Battery in Healthy Adults and Patients With Primary Brain Tumors by S.M. Boelders, E. Butterbrod, L.V.D.E. Vogelsmeier, M.M. Sitskoorn, L.L. Ong and K. Gehring in Assessment

sj-docx-3-asm-10.1177_10731911241289987 – Supplemental material for Factor Structure and Validity of Composite Scores Resulting From a Computerized Cognitive Test Battery in Healthy Adults and Patients With Primary Brain Tumors Supplemental material, sj-docx-3-asm-10.1177_10731911241289987 for Factor Structure and Validity of Composite Scores Resulting From a Computerized Cognitive Test Battery in Healthy Adults and Patients With Primary Brain Tumors by S.M. Boelders, E. Butterbrod, L.V.D.E. Vogelsmeier, M.M. Sitskoorn, L.L. Ong and K. Gehring in Assessment

sj-docx-4-asm-10.1177_10731911241289987 – Supplemental material for Factor Structure and Validity of Composite Scores Resulting From a Computerized Cognitive Test Battery in Healthy Adults and Patients With Primary Brain Tumors Supplemental material, sj-docx-4-asm-10.1177_10731911241289987 for Factor Structure and Validity of Composite Scores Resulting From a Computerized Cognitive Test Battery in Healthy Adults and Patients With Primary Brain Tumors by S.M. Boelders, E. Butterbrod, L.V.D.E. Vogelsmeier, M.M. Sitskoorn, L.L. Ong and K. Gehring in Assessment

## References

[bibr1-10731911241289987] Agelink van Rentergem J. A. de Vent N. R. Schmand B. A. Murre J. M. J. Staaks J. P. C. , ANDI Consortium, & Huizenga H. M . (2020). The factor structure of cognitive functioning in cognitively healthy participants: A meta-analysis and meta-analysis of individual participant data. Neuropsychology Review, 30(1), 51–96. 10.1007/s11065-019-09423-6 PMC7089912 32008158

[bibr2-10731911241289987] Agelink van Rentergem J. A. Vermeulen I. E. Lee Meeuw Kjoe P. R. Schagen S. B . (2021). Computational modeling of neuropsychological test performance to disentangle impaired cognitive processes in cancer patients. JNCI: Journal of the National Cancer Institute, 113(1), 99–102. 10.1093/jnci/djaa039 32239149 PMC7781462

[bibr3-10731911241289987] Ainur A. Sayang M. Jannoo Z. Yap B. (2017). Sample size and non-normality effects on goodness of fit measures in structural equation models. Pertanika Journal of Science & Technology, 25(2), 575–586.

[bibr4-10731911241289987] Auerswald M. Moshagen M. (2019). How to determine the number of factors to retain in exploratory factor analysis: A comparison of extraction methods under realistic conditions. Psychological Methods, 24(4), 468–491. 10.1037/met0000200 30667242

[bibr5-10731911241289987] Barnard J. Rubin D. B. (1999). Small-sample degrees of freedom with multiple imputation. Biometrika. https://doi-org.tilburguniversity.idm.oclc.org/10.1093/biomet/86.4.948

[bibr6-10731911241289987] Beaujean A. (2015). John Carroll’s views on intelligence: Bi-factor vs. higher-order models. Journal of Intelligence, 3(4), 121–136. 10.3390/jintelligence3040121

[bibr7-10731911241289987] Beele P. Boelders S. M. Rutten G.-J. M. De Baene W. Gehring K. (2024). Preoperative executive functioning impairments in patients with a meningioma: Does a frontal location matter? Brain Imaging and Behavior. 10.1007/s11682-024-00886-7 PMC11582180 38722525

[bibr8-10731911241289987] Benson N. Hulac D. M. Kranzler J. H. (2010). Independent examination of the Wechsler adult intelligence scale—Fourth edition (WAIS-IV): What does the WAIS-IV measure? Psychological Assessment, 22(1), 121–130. 10.1037/a0017767 20230158

[bibr9-10731911241289987] Bishara A. J. Hittner J. B. (2015). Reducing Bias and Error in the Correlation Coefficient Due to Nonnormality. Educational and Psychological Measurement, 75(5), 785–804. 10.1177/0013164414557639 29795841 PMC5965513

[bibr10-10731911241289987] Boelders S. M. De Baene W. Postma E. Gehring K. Ong L. L. (2024). Predicting Cognitive Functioning for Patients with a High-Grade Glioma: Evaluating Different Representations of Tumor Location in a Common Space. Neuroinformatics, 22, 329–352. 10.1007/s12021-024-09671-9 38900230 PMC11329426

[bibr11-10731911241289987] Boelders S. M. Gehring K. Postma E. O. Rutten G. J. M. Ong L. L. (2023). Cognitive functioning in untreated glioma patients: The limited predictive value of clinical variables. Neuro-Oncology, 26(4), 670–683. 10.1093/neuonc/noad221 PMC10995520 38039386

[bibr12-10731911241289987] Brooks B. L. Plourde V. Fay-McClymont T. B. MacAllister W. S. Sherman E. M. S. (2019). Factor structure of the CNS vital signs computerized cognitive battery in youth with neurological diagnoses. Child Neuropsychology, 25(7), 980–991. 10.1080/09297049.2019.1569609 30676266

[bibr13-10731911241289987] Brosseau-Liard P. E. Savalei V. (2014). Adjusting incremental fit indices for nonnormality. Multivariate Behavioral Research, 49(5), 460–470. 10.1080/00273171.2014.933697 26732359

[bibr14-10731911241289987] Brosseau-Liard P. E. Savalei V. Li L. (2012). An investigation of the sample performance of two nonnormality corrections for RMSEA. Multivariate Behavioral Research, 47(6), 904–930. 10.1080/00273171.2012.715252 26735008

[bibr15-10731911241289987] Browne M. W. (2001). An overview of analytic rotation in exploratory factor analysis. Multivariate Behavioral Research, 36(1), 111–150. 10.1207/S15327906MBR3601_05

[bibr16-10731911241289987] Butterbrod E. (2021). Heterogeneous cognitive profiles in patients with glioma, meningioma and pituitary adenoma: The multifaceted value of neuropsychological testing in daily neurosurgical practice [Doctoral dissertation, Tilburg University]. Ridderprint.

[bibr17-10731911241289987] Butterbrod E. Synhaeve N. Rutten G.-J. Schwabe I. Gehring K. Sitskoorn M. (2020). Cognitive impairment three months after surgery is an independent predictor of survival time in glioblastoma patients. Journal of Neuro-Oncology, 149(1), 103–111. 10.1007/s11060-020-03577-7 32643066 PMC7452884

[bibr18-10731911241289987] Butterbrod E. Sitskoorn M. Bakker M. Jakobs B. Fleischeuer R. Roijers J. Rutten G.-J. Gehring K. (2021). The APOE ε4 allele in relation to pre- and postsurgical cognitive functioning of patients with primary brain tumors. European Journal of Neurology, 28(5), 1665–1676. 10.1111/ene.14693 33342004 PMC8247965

[bibr19-10731911241289987] Butterbrod E. Bruijn J. Braaksma M. M. Rutten G.-J. M. Tijssen C. C. Hanse M. C. J. Sitskoorn M. M. Gehring K. (2019). Predicting disease progression in high-grade glioma with neuropsychological parameters: The value of personalized longitudinal assessment. Journal of Neuro-Oncology, 144(3), 511–518. 10.1007/s11060-019-03249-1 31342318 PMC6764928

[bibr20-10731911241289987] Canivez G. L. Watkins M. W. (2010). Investigation of the factor structure of the Wechsler adult intelligence scale—Fourth edition (WAIS–IV): Exploratory and higher order factor analyses. Psychological Assessment, 22(4), 827–836. 10.1037/a0020429 20822259

[bibr21-10731911241289987] Cattell R. (2012). The scientific use of factor analysis in behavioral and life sciences. Springer Science & Business Media.

[bibr22-10731911241289987] Chen F. F. (2007). Sensitivity of goodness of fit indexes to lack of measurement invariance. Structural Equation Modeling: A Multidisciplinary Journal, 14(3), 464–504. 10.1080/10705510701301834

[bibr23-10731911241289987] Chen F. F. (2008). What happens if we compare chopsticks with forks? The Impact of making inappropriate comparisons in cross-cultural research. Journal of Personality and Social Psychology, 95(5), 1005–1018. http://dx.doi.org/10.1037/a0013193 18954190 10.1037/a0013193

[bibr24-10731911241289987] CNS Vital Signs. (n.d.). CNS vital signs interpretation guide. https://www.cnsvs.com/WhitePapers/CNSVS-BriefInterpretationGuide.pdf

[bibr25-10731911241289987] Conway M. Fthenaki A. (2003). Disruption of inhibitory control of memory following lesions to the frontal and temporal lobes. Cortex, 39(4–5), 667–686. 10.1016/S0010-9452(08)70859-1 14584548

[bibr26-10731911241289987] De Baene W. Rijnen S. J. M. Gehring K. Meskal I. Rutten G.-J. M. Sitskoorn M. M. (2019). Lesion symptom mapping at the regional level in patients with a meningioma. Neuropsychology, 33(1), 103–110. 10.1037/neu0000490 30475049

[bibr27-10731911241289987] De Baene W. Rutten G. M. Sitskoorn M. M. (2019). Cognitive functioning in glioma patients is related to functional connectivity measures of the non-tumoural hemisphere. European Journal of Neuroscience, 50(12), 3921–3933. 10.1111/ejn.14535 31370107 PMC6972640

[bibr28-10731911241289987] De Roeck E. E. De Deyn P. P. Dierckx E. Engelborghs S . (2019). Brief cognitive screening instruments for early detection of Alzheimer’s disease: A systematic review. Alzheimer’s Research & Therapy, 11(1), 21. 10.1186/s13195-019-0474-3 PMC6396539 30819244

[bibr29-10731911241289987] Erlanger D. M. Kaushik T. Broshek D. Freeman J. Feldman D. Festa J. (2002). Development and validation of a web-based screening tool for monitoring cognitive status. Journal of Head Trauma Rehabilitation, 17(5), 458–476. 10.1097/00001199-200210000-00007 12802255

[bibr30-10731911241289987] Goette W. (2020). Reconsidering the RBANS factor structure: A systematic literature review and meta-analytic factor analysis. Neuropsychology Review, 30(3), 425–442. 10.1007/s11065-020-09447-3 32691281

[bibr31-10731911241289987] Gondar R. Patet G. Schaller K. Meling T. R. (2021). Meningiomas and cognitive impairment after treatment: A systematic and narrative review. Cancers, 13(8), 1846. 10.3390/cancers13081846 33924372 PMC8070481

[bibr32-10731911241289987] Gualtieri C. Johnson L. (2006). Reliability and validity of a computerized neurocognitive test battery, CNS vital signs. Archives of Clinical Neuropsychology, 21(7), 623–643. 10.1016/j.acn.2006.05.007 17014981

[bibr33-10731911241289987] Gualtieri C. T. Hervey A. S. (2015). The structure and meaning of a computerized neurocognitive test battery. Frontiers in Psychological and Behavioral Science, 4, 11.

[bibr34-10731911241289987] Guilford J. P. (1954). Psychometric methods. McGraw-Hill.

[bibr35-10731911241289987] Hair J. F. Anderson R. E. Tatham R. L. Grablowsky B. J. (1979). Multivariate data analysis. Pipe Books.

[bibr36-10731911241289987] Haring L. Mõttus R. Koch K. Trei M. Maron E. (2015). Factorial validity, measurement equivalence and cognitive performance of the Cambridge Neuropsychological Test Automated Battery (CANTAB) between patients with first-episode psychosis and healthy volunteers. Psychological Medicine, 45(9), 1919–1929. 10.1017/S0033291714003018 25544472

[bibr37-10731911241289987] Harman H. H. Jones W. H. (1966). Factor analysis by minimizing residuals (minres). Psychometrika, 31(3), 351–368. 10.1007/BF02289468 5221131

[bibr38-10731911241289987] Hirschfeld G. von Brachel R. (2014). Improving multiple—group confirmatory factor analysis in R—A tutorial in measurement invariance with continuous and ordinal indicators. Practical Assessment, Research, and Evaluation, 19. 10.7275/qazy-2946

[bibr39-10731911241289987] Horn J. L. (1965). A rationale and test for the number of factors in factor analysis. Psychometrika, 30(2), 179–185. 10.1007/BF02289447 14306381

[bibr40-10731911241289987] Hu L. Bentler P. M. (1999). Cutoff criteria for fit indexes in covariance structure analysis: Conventional criteria versus new alternatives. Structural Equation Modeling: A Multidisciplinary Journal, 6(1), 1–55. 10.1080/10705519909540118

[bibr41-10731911241289987] Keine D. Walker J. Q. Kennedy B. K. Sabbagh M. N. (2019). Development, application, and results from a precision-medicine platform that personalizes multi-modal treatment plans for mild Alzheimer’s disease and at-risk individuals. Current Aging Science, 11(3), 173–181. 10.2174/1874609811666181019101430 PMC6388425 30338750

[bibr42-10731911241289987] Kraemer D. J. M. Rosenberg L. M. Thompson-Schill S. L. (2009). The neural correlates of visual and verbal cognitive styles. The Journal of Neuroscience, 29(12), 3792–3798. 10.1523/JNEUROSCI.4635-08.2009 19321775 PMC2697032

[bibr43-10731911241289987] Kyriazos T. A. (2018). Applied psychometrics: Sample size and sample power considerations in Factor Analysis (EFA, CFA) and SEM in general. Psychology, 09(08), 2207–2230. 10.4236/psych.2018.98126

[bibr44-10731911241289987] Langensee L. Mårtensson J. Jönsen A. Zervides K. Bengtsson A. Nystedt J. Cannerfelt B. Nilsson P. Mannfolk P. Lätt J. Rumetshofer T. Sundgren P. C. (2022). Cognitive performance in systemic lupus erythematosus patients: A cross-sectional and longitudinal study. BMC Rheumatology, 6(1), 22. 10.1186/s41927-022-00253-3 35440096 PMC9019974

[bibr45-10731911241289987] Lanou A. J. Mast A. C. Hill B. D. Kim S.-S. Hanaway P. (2023). A randomized, placebo-controlled clinical trial of a novel dietary supplement on standardized CNS vital signs cognitive performance parameters in adults. Journal of Integrative and Complementary Medicine, 29(5), 303–312. 10.1089/jicm.2022.0543 36856456

[bibr46-10731911241289987] Leitgöb H. Seddig D. Asparouhov T. Behr D. Davidov E. De Roover K. Jak S. Meitinger K. Menold N. Muthén B. Rudnev M. Schmidt P. van de Schoot R. (2022). Measurement invariance in the social sciences: Historical development, methodological challenges, state of the art, and future perspectives. Social Science Research, 102805. 10.1016/j.ssresearch.2022.102805 36796989

[bibr47-10731911241289987] Lewis E. D. Apostol M. Langston J. Parker A. Evans M. (2023). A multi-center, open-label exploratory study to assess cognitive function response to lifestyle changes plus supplementation in healthy adults with risk factors associated with cognitive decline. Applied Sciences, 13(5), 2818. 10.3390/app13052818

[bibr48-10731911241289987] Lezak M. D. Howieson D. B. Loring D. W. Hannay H. J. Fischer J. S. (2004). Neuropsychological assessment (4th ed.). Oxford University Press.

[bibr49-10731911241289987] Lonkhuizen P. J. C. Rijnen S. J. M. Linden S. D. Rutten G. M. Gehring K. Sitskoorn M. M. (2019). Subjective cognitive functioning in patients with a meningioma: Its course and association with objective cognitive functioning and psychological symptoms. Psycho-Oncology, 28(8), 1654–1662. 10.1002/pon.5136 31141624 PMC6772142

[bibr50-10731911241289987] Lorenzo-Seva U. ten Berge J. M. F. (2006). Tucker’s congruence coefficient as a meaningful index of factor similarity. Methodology, 2(2), 57–64. 10.1027/1614-2241.2.2.57

[bibr51-10731911241289987] Ma Y. Carlsson C. M. Wahoske M. L. Blazel H. M. Chappell R. J. Johnson S. C. Asthana S. Gleason C. E. (2021). Latent factor structure and measurement invariance of the NIH toolbox cognition battery in an Alzheimer’s disease research sample. Journal of the International Neuropsychological Society, 27(5), 412–425. 10.1017/S1355617720000922 33012297 PMC8108547

[bibr52-10731911241289987] Mathias J. L. Wheaton P. (2007). Changes in attention and information-processing speed following severe traumatic brain injury: A meta-analytic review. Neuropsychology, 21(2), 212–223. 10.1037/0894-4105.21.2.212 17402821

[bibr53-10731911241289987] McNeish D. (2017). Exploratory factor analysis with small samples and missing data. Journal of Personality Assessment, 99(6), 637–652. 10.1080/00223891.2016.1252382 27929657

[bibr54-10731911241289987] McNeish D. Wolf M. G. (2020). Thinking twice about sum scores. Behavior Research Methods, 52(6), 2287–2305. 10.3758/s13428-020-01398-0 32323277

[bibr55-10731911241289987] Meskal I. Gehring K. van der Linden S. D. Rutten G.-J. M. Sitskoorn M. M. (2015). Cognitive improvement in meningioma patients after surgery: Clinical relevance of computerized testing. Journal of Neuro-Oncology, 121(3), 617–625. 10.1007/s11060-014-1679-8 25502961

[bibr56-10731911241289987] Memória C. M. Yassuda M. S. Nakano E. Y. Forlenza O. V. (2014). Contributions of the Computer-Administered Neuropsychological Screen for Mild Cognitive Impairment (CANS-MCI) for the diagnosis of MCI in Brazil. International Psychogeriatrics, 26(9), 1483–1491. 10.1017/S1041610214000726 24806666

[bibr57-10731911241289987] Nassiri V. (2018). On using multiple imputation for exploratory factor analysis of incomplete data. Behavior Research Methods, 17. https://doi.org/10.3758 10.3758/s13428-017-1000-9 29388116

[bibr58-10731911241289987] Niemand T. Mai R. (2018). Flexible cutoff values for fit indices in the evaluation of structural equation models. Journal of the Academy of Marketing Science, 46(6), 1148–1172. 10.1007/s11747-018-0602-9

[bibr59-10731911241289987] Noll K. R. Sullaway C. Ziu M. Weinberg J. S. Wefel J. S. (2015). Relationships between tumor grade and neurocognitive functioning in patients with glioma of the left temporal lobe prior to surgical resection. Neuro-Oncology, 17(4), 580–587. 10.1093/neuonc/nou233 25227126 PMC4483071

[bibr60-10731911241289987] Oliveira M. O. de. Brucki S. M. D. (2014). Computerized Neurocognitive Test (CNT) in mild cognitive impairment and Alzheimer’s disease. Dementia & Neuropsychologia, 8(2), 112–116. 10.1590/S1980-57642014DN82000005 29213891 PMC5619117

[bibr61-10731911241289987] Ory D. T. Mokhtarian P. L. (2010). The impact of non-normality, sample size and estimation technique on goodness-of-fit measures in structural equation modeling: Evidence from ten empirical models of travel behavior. Quality & Quantity, 44(3), 427–445. 10.1007/s11135-008-9215-6

[bibr62-10731911241289987] Papathanasiou A. Messinis L. Georgiou V. L. Papathanasopoulos P. (2014). Cognitive impairment in relapsing remitting and secondary progressive multiple sclerosis patients: Efficacy of a computerized cognitive screening battery. ISRN Neurology, 2014, 1–7. 10.1155/2014/151379 PMC3976849 25006497

[bibr63-10731911241289987] Paul R. K. (2006). Multicollinearity: Causes, effects and remedies. IASRI, 14.

[bibr64-10731911241289987] Picton T. W. Stuss D. T. Alexander M. P. Shallice T. Binns M. A. Gillingham S. (2006). Effects of focal frontal lesions on response inhibition. Cerebral Cortex, 17(4), 826–838. 10.1093/cercor/bhk031 16699079

[bibr65-10731911241289987] Pilipenko P. Ivanova A. A. Kotsiubinskaya Y. V. Feigin V. Majdan M. Grigoryeva V. N. Khrulev A. Y. (2022). Randomised, double-blind, placebo-controlled study investigating Safety and efficAcy of MLC901 in post-traumatic bRAin Injury: The SAMURAI study protocol. BMJ Open, 12(4), Article e059167. 10.1136/bmjopen-2021-059167 PMC9014072 35418437

[bibr66-10731911241289987] Plourde V. Hrabok M. Sherman E. M. S. Brooks B. L. (2018). Validity of a computerized cognitive battery in children and adolescents with neurological diagnoses. Archives of Clinical Neuropsychology, 33(2), 247–253. 10.1093/arclin/acx067 28981565 PMC6093387

[bibr67-10731911241289987] Ratcliff R. McKoon G. (2008). The diffusion decision model: Theory and data for two-choice decision tasks. Neural Computation, 20(4), 873–922. 10.1162/neco.2008.12-06-420 18085991 PMC2474742

[bibr68-10731911241289987] Revelle W. (2017). psych: Procedures for psychological, psychometric, and personality research. Northwestern University. https://CRAN.R-project.org/package=psych

[bibr69-10731911241289987] Rijnen S. (2019). Prediction of cognitive outcome after surgery in patients with meningiomas and gliomas: A comparison with healthy controls using normative formulae and reliable change indices. [Doctoral Thesis, Tilburg University]. [s.n.].

[bibr70-10731911241289987] Rijnen S. J. M. Butterbrod E. Rutten G.-J. M. Sitskoorn M. M. Gehring K. (2020). Presurgical identification of patients with glioblastoma at risk for cognitive impairment at 3-month follow-up. Neurosurgery, Nyaa, 190. 10.1093/neuros/nyaa190 PMC7666888 32470985

[bibr71-10731911241289987] Rijnen S. J. M. Kaya G. Gehring K. Verheul J. B. Wallis O. C. Sitskoorn M. M. Rutten G.-J. M. (2020). Cognitive functioning in patients with low-grade glioma: Effects of hemispheric tumor location and surgical procedure. Journal of Neurosurgery, 133(6), 1671–1682. 10.3171/2019.8.JNS191667 31731264

[bibr72-10731911241289987] Rijnen S. J. M. van der Linden S. D. Emons W. H. M. Sitskoorn M. M. Gehring K. (2018). Test-retest reliability and practice effects of a computerized neuropsychological battery: A solution-oriented approach. Psychological Assessment, 30(12), 1652–1662. 10.1037/pas0000618 29952595

[bibr73-10731911241289987] Rijnen S. J. M. Meskal I. Emons W. H. M. Campman C. A. M. van der Linden S. D. Gehring K. Sitskoorn M. M. (2020). Evaluation of normative data of a widely used computerized neuropsychological battery: Applicability and effects of sociodemographic variables in a dutch sample. Assessment, 27(2), 373–383. 10.1177/1073191117727346 28895436 PMC6990455

[bibr74-10731911241289987] Rosseel Y. (2012). Lavaan: An R package for structural equation modeling. Journal of Statistical Software, 48(2), 1–36. 10.18637/jss.v048.i02

[bibr75-10731911241289987] Satorra A. Bentler P. M. (2001). A scaled difference chi-square test statistic for moment structure analysis. Psychometrika, 66(4), 507–514. 10.1007/BF02296192 PMC2905175 20640194

[bibr76-10731911241289987] Schneider W. J. McGrew K. S. (2018). The Cattell-Horn-Carroll theory of cognitive abilities. Contemporary Intellectual Assessment: Theories, Tests, and Issues, 73–163.

[bibr77-10731911241289987] Schreiber J. B. Nora A. Stage F. K. Barlow E. A. King J. (2006). Reporting structural equation modeling and confirmatory factor analysis results: A review. The Journal of Educational Research, 99(6), 323–338. 10.3200/JOER.99.6.323-338

[bibr78-10731911241289987] Taasoobshirazi G. Wang S. (2016). The performance of the SRMR, RMSEA, CFI, and TLI: An examination of sample size, path size, and degrees of freedom. Quantitative Methods Inquires, 11, 31.

[bibr79-10731911241289987] Tornatore J. B. Hill E. Laboff J. A. McGann M. E. (2005). Self-Administered screening for mild cognitive impairment: Initial validation of a computerized test battery. The Journal of Neuropsychiatry and Clinical Neurosciences, 17(1), 98–105. 10.1176/jnp.17.1.98 15746489 PMC1559991

[bibr80-10731911241289987] Tucker, & Ledyard. (1951). A method for synthesis of factor analysis. Educational Testing Service Princeton NJ.

[bibr81-10731911241289987] van der Linden S. D. Gehring K. De Baene W. Emons W. H. M. Rutten G.-J. M. Sitskoorn M. M. (2020). Assessment of Executive Functioning in Patients with Meningioma and Low-Grade Glioma: A Comparison of Self-Report, Proxy-Report, and Test Performance. Journal of the International Neuropsychological Society, 26(2), 187–196. 10.1017/S1355617719001164 31699166

[bibr82-10731911241289987] van Kessel E. Baumfalk A. E. van Zandvoort M. J. E. Robe P. A. Snijders T. J . (2017). Tumor-related neurocognitive dysfunction in patients with diffuse glioma: A systematic review of neurocognitive functioning prior to anti-tumor treatment. Journal of Neuro-Oncology, 134(1), 9–18. 10.1007/s11060-017-2503-z 28567586 PMC5543199

[bibr83-10731911241289987] van Kessel E. Emons M. A. C. Wajer I. H. van Baarsen K. M. Broekman M. L. Robe P. A. Snijders T. J . (2019). Tumor-related neurocognitive dysfunction in patients with diffuse glioma: A retrospective cohort study prior to antitumor treatment. http://dx.doi.org/10.1093/nop/npz008 10.1093/nop/npz008 PMC6899056 31832216

[bibr84-10731911241289987] van Loenen I. S. Rijnen S. J. M. Bruijn J. Rutten G.-J. M. Gehring K. Sitskoorn M. M. (2018). Group Changes in Cognitive Performance After Surgery Mask Changes in Individual Patients with Glioblastoma. World Neurosurgery, 117, e172–e179. 10.1016/j.wneu.2018.05.232 29886297

[bibr85-10731911241289987] Verhage F. (1965). Intelligence and age in a Dutch sample. Human Development, 8(4), 238–245. 10.1159/000270308

[bibr86-10731911241289987] Watkins M. W. (2018). Exploratory factor analysis: A guide to best practice. Journal of Black Psychology, 44(3), 219–246. 10.1177/0095798418771807

[bibr87-10731911241289987] White C. N. Servant M. Logan G. D. (2018). Testing the validity of conflict drift-diffusion models for use in estimating cognitive processes: A parameter-recovery study. Psychonomic Bulletin & Review, 25(1), 286–301. 10.3758/s13423-017-1271-2 28357629 PMC5788738

[bibr88-10731911241289987] Wicherts J. M. (2016). The importance of measurement invariance in neurocognitive ability testing. The Clinical Neuropsychologist, 30(7), 1006–1016. 10.1080/13854046.2016.1205136 27356958

[bibr89-10731911241289987] Wojcik C. M. Beier M. Costello K. DeLuca J. Feinstein A. Goverover Y. Gudesblatt M. Jaworski M. Kalb R. Kostich L. LaRocca N. G. Rodgers J. D. Benedict R. H. , & National MS Society Cognition Work Team (2019). Computerized neuropsychological assessment devices in multiple sclerosis: A systematic review. Multiple Sclerosis Journal, 25(14), 1848–1869. 10.1177/1352458519879094 31637963 PMC6875828

[bibr90-10731911241289987] Yang K.-C. Hsieh W.-C. Chou Y.-H. (2022). Cognitive factor structure and measurement invariance between healthy controls and patients with major depressive disorder. Journal of Psychiatric Research, 151, 598–605. 10.1016/j.jpsychires.2022.05.029 35636038

[bibr91-10731911241289987] Zygmont C. Smith M. R. (2014). Robust factor analysis in the presence of normality violations, missing data, and outliers: Empirical questions and possible solutions. The Quantitative Methods for Psychology, 10(1), 16. http://dx.doi.org/10.20982/tqmp.10.1.p040

[bibr92-10731911241289987] Zygouris S. Tsolaki M. (2015). Computerized cognitive testing for older adults: A review. American Journal of Alzheimer’s Disease & Other Dementiasr, 30(1), 13–28. 10.1177/1533317514522852 PMC10852880 24526761

