# Supplementary material for: Factor Structure and Validity of Composite Scores Resulting From a Computerized Cognitive Test Battery in Healthy Adults and Patients With Primary Brain Tumors
Source: Assessment. 2024 Nov 20;32(7):1082–102. doi: 10.1177/10731911241289987 (PMC12397561; doi:10.1177/10731911241289987)
Supplement: sj-docx-1-asm-10.1177_10731911241289987 – Supplemental material for Factor Structure and Validity of Composite Scores Resulting From a Computerized Cognitive Test Battery in Healthy Adults and Patients With Primary Brain Tumors [file sj-docx-1-asm-10.1177_10731911241289987.docx]

Table S1: Descriptions and resulting variables from the seven core tests of CNS VS

| Test name | Description | Resulting variables |
| --- | --- | --- |
| Verbal memory | Fifteen words are presented, one at a time. Participant subsequently identifies presented words among 15 new (distractor) words, presented one at a time by pressing the space bar. Contains both an initial and delayed condition. | - Initial condition; number of correct hits - Initial condition; number of correct passes - Delayed condition; number of correct hits - Delayed condition; number of correct passes |
| Visual memory | Fifteen abstract line images are presented, one at a time. Participant subsequently identifies presented images among 15 new (distractor) images, presented one at a time by pressing the space bar. Contains both an initial and delayed condition. | - Initial condition; correct hits - Initial condition; correct passes - Delayed condition; correct hits - Delayed condition; correct passes |
| Symbol digit coding | Participant types in the number (from 2 through 9) on the number row that corresponds with a symbol that is highlighted. Fixed test time of two minutes. | - Number of correct hits - Number of errors |
| Stroop test | Part 1 (simple): one word (describing a color) is presented at a time in black font. Participant presses space bar when as soon as the word is presented. Part 2 (congruent): Participant presses the space bar if the color of the word matches the meaning of the word.  Part 3 (incongruent): Participant presses the space bar if the color of the word does not match the meaning of the word. | - Simple, reaction time - Congruent, correct hits - Congruent, correct passes - Congruent, reaction time on correct hits - Incongruent, correct hits - Incongruent, correct passes - Incongruent, reaction time on correct hits |
| Continuous performance test | The test subject is asked to respond to the target stimulus “B” but not to any other letter. In five minutes, 40 B’s are randomly presented among 160 distractor letters. Presentation times are variable throughout the test. The test takes around 5 minutes to complete. | - Reaction time - Number of correct hits - Number of omission errors (exact inverse of correct hits) |
| Shifting attention task | Three figures appear on the screen, one on top and two on the bottom. The top figure is either a square or a circle, in either red or blue color. The bottom figures (one presented on bottom left and one on the bottom right of the screen) are a square or a circle, and red or blue. The participant is asked to match one of the bottom figures to the top shape, or match one of the bottom colors to the top color. The rules change at random with each new item (i.e., match the figures by shape, or by color). Fixed test time of two minutes. | - Number of correct hits - Number of errors (co-linear with number of correct hits) - Reaction time on correct hits |
| Finger tapping test | Participant presses space bar as quickly as possible for 10 seconds (index finger, three trails per side). | - Average number of left taps - Average number of right taps |

Please refer to: [Microsoft PowerPoint - CNS_VS_Brief_Interpretation_Guide (cnsvs.com)](https://www.cnsvs.com/WhitePapers/CNSVS-BriefInterpretationGuide.pdf)
