# Supplementary material for: Factor Structure and Validity of Composite Scores Resulting From a Computerized Cognitive Test Battery in Healthy Adults and Patients With Primary Brain Tumors
Source: Assessment. 2024 Nov 20;32(7):1082–102. doi: 10.1177/10731911241289987 (PMC12397561; doi:10.1177/10731911241289987)
Supplement: sj-docx-2-asm-10.1177_10731911241289987 – Supplemental material for Factor Structure and Validity of Composite Scores Resulting From a Computerized Cognitive Test Battery in Healthy Adults and Patients With Primary Brain Tumors [file sj-docx-2-asm-10.1177_10731911241289987.docx]

Table S2: Fit measures for 20 confirmatory models, established individually for each group and number of factors maintained

| Number of factors | Sample used for the EFA and CFA | Chi-square | Degrees of freedom | | p-value | | CFI | | RMSEA | SRMR |
| --- | --- | --- | --- | --- | --- | --- | --- | --- | --- | --- |
| 2 | Healthy participants | 443.85 | | 251 | | 0.000 | | 0.657 | 0.071 | 0.095 |
|  | Meningioma | 997.06 | | 251 | | 0.000 | | 0.649 | 0.100 | 0.114 |
|  | Low-grade glioma | 467.93 | | 251 | | 0.000 | | 0.539 | 0.108 | 0.123 |
|  | High-grade glioma | 730.58 | | 251 | | 0.000 | | 0.619 | 0.101 | 0.130 |
| 3 | Healthy participants | 396.76 | | 249 | | 0.000 | | 0.744 | 0.061 | 0.084 |
|  | Meningioma | 877.74 | | 249 | | 0.000 | | 0.709 | 0.091 | 0.098 |
|  | Low-grade glioma | 454.78 | | 249 | | 0.000 | | 0.577 | 0.104 | 0.125 |
|  | High-grade glioma | 700.12 | | 249 | | 0.000 | | 0.657 | 0.096 | 0.123 |
| 4 | Healthy participants | 337.30 | | 246 | | 0.000 | | 0.841 | **0.049** | 0.081 |
|  | Meningioma | 735.37 | | 246 | | 0.000 | | 0.773 | 0.081 | 0.098 |
|  | Low-grade glioma | 401.12 | | 246 | | 0.000 | | 0.681 | 0.091 | 0.106 |
|  | High-grade glioma | 650.32 | | 246 | | 0.000 | | 0.703 | 0.090 | 0.104 |
| 5 | Healthy participants | 291.46 | | 242 | | 0.016 | | 0.914 | **0.036** | 0.076 |
|  | Meningioma | 599.82 | | 242 | | 0.000 | | 0.834 | 0.070 | 0.081 |
|  | Low-grade glioma | 374.64 | | 242 | | 0.000 | | 0.729 | 0.085 | 0.105 |
|  | High-grade glioma | 585.32 | | 242 | | 0.000 | | 0.744 | 0.084 | 0.111 |
| 6 | Healthy participants | 255.38 | | 237 | | 0.197 | | 0.968 | **0.022** | 0.068 |
|  | Meningioma | 535.60 | | 237 | | 0.000 | | 0.860 | 0.065 | 0.079 |
|  | Low-grade glioma | 352.58 | | 237 | | 0.000 | | 0.761 | 0.080 | 0.103 |
|  | High-grade glioma | 522.06 | | 237 | | 0.000 | | 0.787 | 0.077 | 0.104 |

Fit measures of 20 different confirmatory models established using exploratory factor analysis, individually for each combination of group and numbers of factors maintained. RMSEA Metrics indicating good model fit are displayed in bold. EFA: Exploratory factor analysis, CFA: Confirmatory factor analysis, CFI: Comparative Fit Index, RMSEA: Root Mean Square Error of Approximation, SRMR: standardized root mean square residual.
