# Supplementary material for: Factor Structure and Validity of Composite Scores Resulting From a Computerized Cognitive Test Battery in Healthy Adults and Patients With Primary Brain Tumors
Source: Assessment. 2024 Nov 20;32(7):1082–102. doi: 10.1177/10731911241289987 (PMC12397561; doi:10.1177/10731911241289987)
Supplement: sj-docx-3-asm-10.1177_10731911241289987 – Supplemental material for Factor Structure and Validity of Composite Scores Resulting From a Computerized Cognitive Test Battery in Healthy Adults and Patients With Primary Brain Tumors [file sj-docx-3-asm-10.1177_10731911241289987.docx]

Table S3: Fit measures for 5 confirmatory models

| Number of factors | Sample | chi-square | Degrees of freedom | p-value | CFI | RMSEA | SRMR |
| --- | --- | --- | --- | --- | --- | --- | --- |
| 1 | Meningioma | 1289.84 | 252 | 0.00 | 0.51 | 0.12 | 0.13 |
|  | Low-grade glioma | 579.09 | 252 | 0.00 | 0.33 | 0.13 | 0.15 |
|  | High-grade glioma | 894.41 | 252 | 0.00 | 0.51 | 0.11 | 0.12 |
|  | Healthy participants | 712.23 | 252 | 0.00 | 0.80 | 0.08 | 0.08 |
|  | All patients (MG-CFA) | 1597.12 | 756.00 | 0.00 | 0.48 | 0.12 | 0.14 |
| 2 | Meningioma | 997.06 | 251 | 0.00 | 0.65 | 0.10 | 0.11 |
|  | Low-grade glioma | 538.61 | 251 | 0.00 | 0.38 | 0.13 | 0.15 |
|  | High-grade glioma | 731.94 | 251 | 0.00 | 0.62 | 0.10 | 0.13 |
|  | Healthy participants | 442.27 | 251 | 0.00 | 0.66 | 0.07 | 0.09 |
|  | All patients (MG-CFA) | 1376.38 | 753.00 | 0.00 | 0.60 | 0.10 | 0.14 |
| 3 | Meningioma | 917.21 | 249 | 0.00 | 0.69 | 0.09 | 0.11 |
|  | Low-grade glioma | 603.77 | 249 | 0.00 | 0.24 | 0.14 | 0.17 |
|  | High-grade glioma | 700.12 | 249 | 0.00 | 0.66 | 0.10 | 0.12 |
|  | Healthy participants | 437.06 | 249 | 0.00 | 0.67 | 0.07 | 0.09 |
|  | All patients (MG-CFA) | 1326.06 | 747.00 | 0.00 | 0.64 | 0.10 | 0.14 |
| 4 | Meningioma | 738.93 | 246 | 0.00 | 0.77 | 0.08 | 0.08 |
|  | Low-grade glioma | 416.99 | 246 | 0.00 | 0.64 | 0.10 | 0.12 |
|  | High-grade glioma | 588.73 | 246 | 0.00 | 0.75 | 0.08 | 0.09 |
|  | Healthy participants | 391.57 | 246 | 0.00 | 0.75 | *0.06 | 0.08 |
|  | All patients (MG-CFA) | 1172.07 | 738.00 | 0.00 | 0.73 | 0.09 | 0.10 |
| 5 | Meningioma | 580.65 | 242 | 0.00 | 0.84 | 0.07 | 0.08 |
|  | Low-grade glioma | 386.97 | 242 | 0.00 | 0.70 | 0.09 | 0.11 |
|  | High-grade glioma | 527.56 | 242 | 0.00 | 0.79 | 0.08 | 0.08 |
|  | Healthy participants | 417.47 | 242 | 0.00 | 0.69 | 0.07 | 0.09 |
|  | All patients (MG-CFA) | 1070.59 | 726.00 | 0.00 | 0.79 | 0.08 | 0.10 |

Appendix 4: Fit measures of five different confirmatory models established using exploratory factor analysis on the balanced sample comprising all patient groups (one model per number of factors maintained). The confirmatory factor analysis on the balanced sample consisting of all patient groups was performed using a multi-group confirmatory factor analysis (MG-CFA). None of the measures indicated good model fit. CFI: Comparative Fit Index, RMSEA: Root Mean Square Error of Approximation, SRMR: standardized root mean square residual. *Rounded down from 0.061.
