# Supplementary material for: Factor Structure and Validity of Composite Scores Resulting From a Computerized Cognitive Test Battery in Healthy Adults and Patients With Primary Brain Tumors
Source: Assessment. 2024 Nov 20;32(7):1082–102. doi: 10.1177/10731911241289987 (PMC12397561; doi:10.1177/10731911241289987)
Supplement: sj-docx-4-asm-10.1177_10731911241289987 – Supplemental material for Factor Structure and Validity of Composite Scores Resulting From a Computerized Cognitive Test Battery in Healthy Adults and Patients With Primary Brain Tumors [file sj-docx-4-asm-10.1177_10731911241289987.docx]

Table S4: Tucker's congruence coefficients

| LGG \ MEN | Attention | Memory correct passes / Strategy | Recognition of visual and verbal material | Inhibition | Motor speed |
| --- | --- | --- | --- | --- | --- |
| Attention | 0.84 | 0.15 | -0.05 | -0.04 | 0.21 |
| Psychomotor speed | 0.45 | 0.35 | 0.11 | -0.18 | 0.42 |
| Inhibition | 0.29 | 0.07 | 0.08 | 0.74 | 0.41 |
| Recognition of visual and verbal material | 0.07 | -0.23 | **0.91** | 0.06 | 0.21 |
| Memory correct passes | 0.07 | 0.81 | -0.04 | 0.34 | -0.03 |
|  |  |  |  |  |  |
| HGG \ MEN | Attention | Memory correct passes / Strategy | Recognition of visual and verbal material | Inhibition | Motor speed |
| Attention | **0.93** | 0.12 | 0.20 | 0.04 | 0.16 |
| Memory correct passes / Strategy | 0.07 | **0.92** | -0.3 | 0 | -0.04 |
| Inhibition | 0.3 | 0.13 | 0.02 | **0.86** | 0.17 |
| Recognition of verbal material | 0.1 | -0.07 | **0.86** | -0.15 | 0.02 |
| Motor speed | 0.1 | -0.01 | 0.05 | 0.12 | **0.89** |
|  |  |  |  |  |  |
| HGG \ LGG | Attention | Psychomotor speed | Inhibition | Recognition of visual and verbal material | Memory correct passes |
| Attention | **0.85** | 0.43 | 0.21 | 0.21 | 0.12 |
| Memory correctpasses / Strategy | 0.16 | 0.31 | 0.01 | -0.37 | 0.7 |
| Inhibition | 0.07 | 0.09 | 0.83 | -0.01 | 0.23 |
| Recognition of verbal material | -0.03 | 0.14 | -0.03 | 0.74 | 0.02 |
| Motor speed | 0.27 | 0.24 | 0.41 | 0.23 | -0.07 |
|  |  |  |  |  |  |
| HP \ MEN | Attention | Memory correct passes / Strategy | Recognition of visual and verbal material | Inhibition | Motor speed |
| Information processing speed | 0.76 | -0.02 | 0.04 | -0.36 | 0.15 |
| General cognitive performance | 0.37 | 0.76 | 0.04 | 0.36 | 0.12 |
| Recognition of verbal material | -0.04 | 0.02 | 0.82 | 0.02 | 0.02 |
| Motor speed | 0.21 | -0.06 | 0.02 | 0.06 | 0.87 |
| Recognition of visual material | 0.15 | -0.44 | 0.52 | -0.02 | 0.07 |
|  |  |  |  |  |  |
| HP \ LGG | Attention | Psychomotor speed | Inhibition | Recognition of visual and verbal material | Memory correct passes |
| Information processing speed | 0.8 | 0.41 | -0.16 | -0.01 | -0.06 |
| General cognitive performance | 0.25 | 0.61 | 0.29 | -0.02 | 0.67 |
| Recognition of verbal material | -0.06 | -0.01 | 0.03 | 0.70 | 0.2 |
| Motor speed | 0.22 | 0.38 | 0.43 | 0.17 | -0.09 |
| Recognition of visual material | 0.06 | 0.03 | -0.03 | 0.65 | -0.32 |
|  |  |  |  |  |  |
| HP \ HGG | Attention | Memory correct passes / Strategy | Inhibition | Recognition of verbal material | Motor speed |
| Information processing speed | 0.76 | 0.03 | -0.23 | 0.06 | 0.11 |
| General cognitive performance | 0.37 | 0.59 | 0.48 | 0.06 | 0.02 |
| Recognition of verbal material | -0.01 | 0 | 0 | 0.84 | 0.09 |
| Motor speed | 0.14 | -0.01 | 0.24 | 0.09 | 0.78 |
| Recognition of visual material | 0.31 | -0.68 | -0.05 | 0.27 | -0.01 |
|  |  |  |  |  |  |

Tucker's congruence coefficient between each pair of factors. Scores indicating fair similarity (between 0.85 and 0.94) are indicated in bold. Scores indicating good similarity (>0.95) are underlined. LGG: Low-grade glioma, HGG: High-grade glioma, MEN: Meningioma, HP: Healthy participants
